# Supplementary material for: Safety, effectiveness and immunogenicity of heterologous mRNA-1273 boost after prime with Ad26.COV2.S among healthcare workers in South Africa: The single-arm, open-label, phase 3 SHERPA study
Source: PLOS Glob Public Health. 2024 Dec 5;4(12):e0003260. doi: 10.1371/journal.pgph.0003260 (PMC11620404; doi:10.1371/journal.pgph.0003260)
Supplement: S1 Table — (DOCX) [file pgph.0003260.s002.docx]

**Supplementary Table 1: Study schema of Sisonke mRNA-1273 boost and comparator groups**

| **Study Groups** | **Sisonke**  **(Feb–May 2021)** | **Sisonke 2**  **(Nov-Dec 2021)** | **mRNA boost**  **(SHERPA May-Nov 2022)** |
| --- | --- | --- | --- |
| mRNA boost  Group 1 | Ad26.COV2.S | Ad26.COV2.S | mRNA-1273 |
| mRNA boost  Group 2 | Ad26.COV2.S | X | mRNA-1273 |
| Sisonke Comparator Group 1 | Ad26.COV2.S | Ad26.COV2.S | X |
| Sisonke Comparator Group 2 | Ad26.COV2.S | X | X |
