# Supplementary material for: Safety, effectiveness and immunogenicity of heterologous mRNA-1273 boost after prime with Ad26.COV2.S among healthcare workers in South Africa: The single-arm, open-label, phase 3 SHERPA study
Source: PLOS Glob Public Health. 2024 Dec 5;4(12):e0003260. doi: 10.1371/journal.pgph.0003260 (PMC11620404; doi:10.1371/journal.pgph.0003260)
Supplement: S2 Table — (DOCX) [file pgph.0003260.s003.docx]

**Supplementary Table 2: Eligibility criteria for the SHERPA trial**

| **Inclusion criteria** |
| --- |
| - Age 18 and older at time of enrolment - Sisonke participant - Received a priming Ad26.CoV2.S vaccination as part of the Sisonke study - If received a second dose of Ad26.CoV2.S vaccine as part of the Sisonke 2 study, this was administered at least 3 months ago - Participants who are pregnant or report breastfeeding at the time of enrolment may be included. - Willingness and ability to comply with vaccination plan and other study procedures. - Capable and willing to provide informed consent |
| **Exclusion criteria** |
| - Participants who have received any COVID-19 vaccines other than one or two doses of Ad26.CoV2.S through other means (for example, another mRNA booster dose). - Current participation in any other research studies (other than Sisonke) that would interfere with the objectives of this study. The determination of whether participation in another study would be exclusionary for a given participant will be made by the PI/designee. - Occurrence of a known COVID-19 outcome within 14 days of enrollment - Participants with a history of heparin-induced thrombocytopenia, or thrombosis and thrombocytopenia syndrome. - History of severe adverse reaction associated with a vaccine and/or severe allergic reaction (e.g., anaphylaxis) to any component of mRNA-1273. - Pregnancy which began prior to or within 30 days after the receipt of dose 2 of Ad26.COV2 and ongoing at the time of enrolment in this trial. - Acute infection (e.g. febrile illness) unless resolved before enrolment. - Any significant acute or chronic medical condition, situation or circumstance that in the opinion of the PI/designee makes the participant unsuitable for participation in the study, or jeopardises the safety or rights of the participant |
| **The following participants should only be enrolled after discussion with the PSRT** |
| - Participants who are thought to have suffered a neurological or cardiac adverse event considered related to the Ad26.CoV2.S vaccine. - Participants reporting a non-infective serious adverse event within the first 28 days following the first or second dose of Ad26.CoV2.S vaccine in the Sisonke trial - History of myocarditis or pericarditis especially in young male - Chronic history of severe clotting disorders - Participants who suffered a thromboembolic adverse event following the Ad26.CoV2.S vaccine. |
