# Supplementary material for: Safety, effectiveness and immunogenicity of heterologous mRNA-1273 boost after prime with Ad26.COV2.S among healthcare workers in South Africa: The single-arm, open-label, phase 3 SHERPA study
Source: PLOS Glob Public Health. 2024 Dec 5;4(12):e0003260. doi: 10.1371/journal.pgph.0003260 (PMC11620404; doi:10.1371/journal.pgph.0003260)
Supplement: S3 Table — (DOCX) [file pgph.0003260.s004.docx]

**Supplementary Table 3: Screening and enrolment visit procedures in the SHERPA study**

| **Heterologous mRNA-1273 Boost Study (Main cohort nested in Sisonke)** | | |
| --- | --- | --- |
| **Visit Number** | **1** | **2** |
| **Study Day** | **-56 to 1** | **1** |
| **Procedure** | **Screen** | **Vaccine** |
| **Study procedures** |  |  |
| Assessment of Understanding | √ |  |
| Informed consent | √ |  |
| Medical history | √ |  |
| Vaccination history | √ |  |
| Physical exam | √ |  |
| Obtain demographics | √ |  |
| Concomitant medications | √ |  |
| Vaccination |  | √ |
| SAEs, AESIs |  | √ |
| **Specimen Collections** |  |  |
| Pregnancy test^#^ | √ |  |
| Blood plasma for SARS CoV-2 serology (4 mls EDTA) |  | √ |
| Nasal swab for SARS CoV-2 PCR |  | √ |

# Female participants only. Pregnant women were added to the pregnancy registry of the study and had an obstetric and gynaecological history recorded. Follow up was conducted through the sub-study and through the central Sisonke safety desk.
