# Supplementary material for: Safety, effectiveness and immunogenicity of heterologous mRNA-1273 boost after prime with Ad26.COV2.S among healthcare workers in South Africa: The single-arm, open-label, phase 3 SHERPA study
Source: PLOS Glob Public Health. 2024 Dec 5;4(12):e0003260. doi: 10.1371/journal.pgph.0003260 (PMC11620404; doi:10.1371/journal.pgph.0003260)
Supplement: S4 Table — (DOCX) [file pgph.0003260.s005.docx]

**Supplementary Table 4: Schedule of evaluation for participants in the safety and immunogenicity sub-study (approximately N=200)**

| **Safety and Immunogenicity Sub-study** | | |  |  |
| --- | --- | --- | --- | --- |
| **Visit Number** | **1** | **2** | **3** | **4** |
| **Study Week** |  | 0 | 4 | 24 |
| **Study Day** | -56 to 1 | 1 | 29 | 169 |
| **Procedure** | **Screen** | **Vaccine** |  |  |
| **Study procedures** |  |  |  |  |
| Assessment of Understanding | √ |  |  |  |
| Informed consent | √ |  |  |  |
| Medical history | √ |  |  |  |
| Gynaecological and obstetric history* | (√) |  |  |  |
| Vaccination history | √ |  |  |  |
| Physical exam | √ |  |  |  |
| Obtain demographics | √ |  |  |  |
| Concomitant medications | √ |  |  |  |
| Vaccination |  | √ |  |  |
| SAEs, AESIs assessment |  | √ | √ | √ |
| Early reactogenicity assessment |  | √ |  |  |
| AEs for 28 days post vaccination |  | √ | √ |  |
| **Specimen Collections** |  |  |  |  |
| Pregnancy test ^#^ | √ |  |  |  |
| Blood plasma (approx. 8 mls) |  | √ | √ | √ |
| Blood PBMC (approx. 42 mls)^+^ |  | √ | √ | √ |
| Nasal swab for COVID PCR |  | √ | √ | √ |
| Breastmilk^ for immune responses |  | (√) | (√) | (√) |

^#^female participants only, *pregnant and breastfeeding participants only, ^optional procedure for breastfeeding women, ^+^no peripheral blood mononuclear cells (PBMC) samples were collected from pregnant women
