# Supplementary material for: Safety, effectiveness and immunogenicity of heterologous mRNA-1273 boost after prime with Ad26.COV2.S among healthcare workers in South Africa: The single-arm, open-label, phase 3 SHERPA study
Source: PLOS Glob Public Health. 2024 Dec 5;4(12):e0003260. doi: 10.1371/journal.pgph.0003260 (PMC11620404; doi:10.1371/journal.pgph.0003260)
Supplement: S5 Table — (DOCX) [file pgph.0003260.s006.docx]

**Supplementary Table 5: Number of SARS-CoV-2 infection and severe Covid-19 events and total exposure time (in years) among SHERPA and non-SHERPA Sisonke participants**

|  | **SHERPA** | | | **non-SHERPA** | | |
| --- | --- | --- | --- | --- | --- | --- |
| **Characteristic** | **Ad26.COV2.S + mRNA-1273** | **2 Ad26.COV2.S + mRNA-1273** | **Total** | **Ad26.COV2.S** | **2 Ad26.COV2.S** | **Total** |
| **SARS-CoV-2 infections** | | | | | | |
| After SHERPA start, but before mRNA-1273 booster | 6 | 13 | 19 | 905 | 2447 | **3352** |
| After mRNA-1273 booster | **2** | **11** | **13** |  |  |  |
| Total | 8 | 24 | 32 |  |  |  |
| Days to infection after mRNA 1273 booster*  Median, IQR | 113.5  (100-127) | 125  (47-164) | 125  (90-154) |  |  |  |
| **Person years at risk#** | | | | | | |
| After SHERPA but before mRNA-1273 booster | 1374.28 | 1682.71 | 3056.99 | 113092.52 | 138012.43 | **251104.95** |
| **After mRNA-1273 booster** | **1553.90** | **1843.96** | **3397.87** |  |  |  |
| **Severe endpoint (COVID-19 hospitalizations or death)** | | | | | | |
| After SHERPA start, but before mRNA-1273 booster | 0 | 0 | 0 | 53 | 95 | **148** |
| After mRNA-1273 booster | 0 | 1 | 1 |  |  |  |
| Total | **0** | **1** | **1** |  |  |  |
| **Person years at risk#** | | | | | | |
| After SHERPA but before mRNA-1273 booster | 1375.42 | 1686.16 | 3061.58 | 113422.48 | 138947.34 | **252369.82** |
| **After mRNA-1273 booster** | **1555.98** | **1848.68** | **3404.66** |  |  |  |

*Events were counted from 14 days after the mRNA-1273 booster. The earliest time an infection occurred in SHERPA was 30 days after vaccination.

#Since the two study groups had dynamic membership and participants could contribute data to both groups, the table presents the percentage of person-years at risk rather than the number of individual participants. SHERPA participants initially included in the non-booster exposure period and left it after receipt of mRNA-1273, provided they did not have confirmed infection.
