# Supplementary material for: Safety, effectiveness and immunogenicity of heterologous mRNA-1273 boost after prime with Ad26.COV2.S among healthcare workers in South Africa: The single-arm, open-label, phase 3 SHERPA study
Source: PLOS Glob Public Health. 2024 Dec 5;4(12):e0003260. doi: 10.1371/journal.pgph.0003260 (PMC11620404; doi:10.1371/journal.pgph.0003260)
Supplement: S6 Table — (DOCX) [file pgph.0003260.s007.docx]

**Supplementary Table 6: Relative vaccine effectiveness of the mRNA-1273 booster against COVID-19 hospitalizations or death estimates (149 adjudicated endpoints)**

|  | **Unadjusted** | **Adjusted*: Model with comorbidities** | **Adjusted*: Model with HIV** |
| --- | --- | --- | --- |
|  | **VE (95% CI)** | **VE (95% CI)** | **VE (95% CI)** |
| **mRNA-1273 boosted**  **group vs not boosted** | 36%  (-358% to 91%) | 42%  (-319% to 92%) | 38%  (-346% to 91%) |

*Adjusted for age, sex, prior vaccination, prior COVID-19, geographical location
