# Supplementary material for: Safety, effectiveness and immunogenicity of heterologous mRNA-1273 boost after prime with Ad26.COV2.S among healthcare workers in South Africa: The single-arm, open-label, phase 3 SHERPA study
Source: PLOS Glob Public Health. 2024 Dec 5;4(12):e0003260. doi: 10.1371/journal.pgph.0003260 (PMC11620404; doi:10.1371/journal.pgph.0003260)
Supplement: S7 Table — (DOCX) [file pgph.0003260.s008.docx]

**Supplementary Table 7: Matched cohort analysis matching SHERPA and non-SHERPA participants 1:1 on key variables**

| **Characteristic** | **SHERPA**  **(N=10 503)** | **NON SHERPA**  **(N=10 502)** |
| --- | --- | --- |
| Sex, no.(%) |  |  |
| female | 8445 (80.4) | 8444 (80.4) |
| male | 2058 (19.6) | 2058 (19.6) |
| Median age(IQR), years | 41 (34 - 48) | 41 (34 - 48) |
| Age groups (year), no.(%) | |  |
| 18-39 | 4511 (42.9) | 4511 (42.9) |
| 40-49 | 3815 (36.3) | 3814 (36.3) |
| 50-59 | 1824 (17.4) | 1825 (17.4) |
| 60+ | 353 (3.4) | 353 (3.4) |
| Number of comorbidities, no.(%) | |  |
| 0 | 7332 (69.8) | 7330 (69.8) |
| 1 | 2802 (26.7) | 2803 (26.7) |
| 2+ | 369 (3.5) | 369 (3.5) |
| HIV infection, no.(%) | 1823 (17.4) | 1273 (12.1) |
| Hypertension, no.(%) | 1237 (11.8) | 1592 (15.2) |
| Diabetes mellitus, no.(%) | 437 (4.2) | 557 (5.3) |
| Cancer, no.(%) | 16 (0.2) | 27 (0.3) |
| Tuberculosis, no.(%) | 13 (0.1) | 15 (0.1) |
| Heart disease, no.(%) | 29 (0.3) | 59 (0.6) |
| Chronic lung disease, no.(%) | 15 (0.1) | 43 (0.4) |
| Geographical location, no.(%) |  |  |
| Eastern Cape | 1512 (14.4) | 1513 (14.4) |
| Free State | 446 (4.2) | 446 (4.3) |
| Gauteng | 3544 (33.7) | 3542 (33.7) |
| KwaZulu-Natal | 2611 (24.9) | 2611 (24.9) |
| Limpopo | 138 (1.3) | 138 (1.3) |
| Mpumalanga | 363 (3.5) | 363 (3.5) |
| North West | 337 (3.2) | 337 (3.2) |
| Northern Cape | 4 (0.0) | 4 (0.0) |
| Western Cape | 1548 (14.7) | 1548 (14.7) |
