# Supplementary material for: Safety, effectiveness and immunogenicity of heterologous mRNA-1273 boost after prime with Ad26.COV2.S among healthcare workers in South Africa: The single-arm, open-label, phase 3 SHERPA study
Source: PLOS Glob Public Health. 2024 Dec 5;4(12):e0003260. doi: 10.1371/journal.pgph.0003260 (PMC11620404; doi:10.1371/journal.pgph.0003260)
Supplement: S8 Table — (DOCX) [file pgph.0003260.s009.docx]

**Supplementary Table 8: Relative Vaccine Effectiveness of the mRNA-1273 booster using the matched cohort analysis approach**

| **SHERPA** | **Non-SHERPA** |  |
| --- | --- | --- |
| **Events/Person Years** | **Events/Person Years** | **Relative Vaccine Effectiveness** |
| 12/3140 | 33/3125 | 63% (32%- 82%) |
