# Supplementary material for: Safety, effectiveness and immunogenicity of heterologous mRNA-1273 boost after prime with Ad26.COV2.S among healthcare workers in South Africa: The single-arm, open-label, phase 3 SHERPA study
Source: PLOS Glob Public Health. 2024 Dec 5;4(12):e0003260. doi: 10.1371/journal.pgph.0003260 (PMC11620404; doi:10.1371/journal.pgph.0003260)
Supplement: S9 Table — (DOCX) [file pgph.0003260.s010.docx]

**Supplementary Table 9: Multivariable logistic regression model** **of local/systemic reactions adjusted for age and sex**

|  | n (%) reporting AE/reactogenicity | Unadjusted OR  (95% CI) | Adjusted OR  (95% CI) |
| --- | --- | --- | --- |
| **Prior COVID diagnosis** | | | |
| No | 147/ 8372 (1.8%) | Reference |  |
| Yes | 124/ 3424 (3.6%) | 2.10 (1.65 - 2.67) | 2.03 (1.59 - 2.59) |
| **HIV status** | | | |
| No | 234/ 8761 (2.7%) | Reference |  |
| Yes | 37/ 2966 (1.2%) | 0.46 (0.32 - 0.65) | 0.49 (0.34 - 0.69) |
| **Prior vaccination** | | | |
| 1 Ad26.COV2.S | 108/ 5436 (2.0%) | Reference |  |
| 2 Ad26.COV2.S | 163/ 6361 (2.6%) | 1.30 (1.01 - 1.66) | 1.26 (0.99 - 1.62) |
