# Supplementary material for: Safety, effectiveness and immunogenicity of heterologous mRNA-1273 boost after prime with Ad26.COV2.S among healthcare workers in South Africa: The single-arm, open-label, phase 3 SHERPA study
Source: PLOS Glob Public Health. 2024 Dec 5;4(12):e0003260. doi: 10.1371/journal.pgph.0003260 (PMC11620404; doi:10.1371/journal.pgph.0003260)
Supplement: S10 Table — (DOCX) [file pgph.0003260.s011.docx]

**Supplementary Table 10: Adverse Pregnancy Outcomes among pregnant Women**

| Age range | Time to Onset (days) | Method of Estimation | Outcome | Estimated Gestational Age at Outcome | Delivery Method | Attribution |
| --- | --- | --- | --- | --- | --- | --- |
| 30-34 | 28 | Clinical exam at outcome, LNMP unsure | Ectopic pregnancy | 12 weeks | N/A | Not Related |
| 20-24 | 32 | Date of LNMP | Miscarriage (< 20 weeks) | 12 weeks 1 day | N/A | Not Related |
| 30-34 | 26 | Clinical exam at outcome, LNMP unsure | Miscarriage (< 20 weeks) | 8 weeks 5 days | N/A | Not Related |
| 35-39 | 28 | Date of LNMP | Miscarriage (< 20 weeks) | 14 weeks | N/A | Not Related |
| 44-49 | 6 | Date of LNMP | Miscarriage (< 20 weeks) | 5 weeks | N/A | Not Related |
| 35-39 | 7 | Clinical examination | Miscarriage (< 20 weeks) | 5 weeks | N/A | Not Related |
| 35-39 | 44 | Date of LNMP | Miscarriage (< 20 weeks) | 10 weeks | N/A | Not Related |
| 30-34 | 70 | Date of LNMP | Miscarriage (< 20 weeks) | 14 weeks | N/A | Not Related |
| 30-34 | 56 | Date of LNMP | Miscarriage (< 20 weeks) | 8 weeks | N/A | Not Related |
| 35-39 | 70 | Date of LNMP | Miscarriage (< 20 weeks) | 14 weeks | N/A | Not Related |
| 30-34 | 41 | Clinical examination | Miscarriage (< 20 weeks) | 5 weeks 6 days | N/A | Not Related |
| 30-34 | Not yet pregnant | Date of LNMP | Miscarriage (< 20 weeks) | ≈12 weeks | N/A | Not Related |
| 30-34 | 177 | Date of LNMP | Premature live birth (< 37 weeks) | 31 weeks | C-section | Not Related |
| 25-29 | 19 | Ultrasound | Premature live birth (< 37 weeks) | 29 weeks | C-section | Not Related |
| 35-39 | 82 | Date of LNMP | Premature live birth (< 37 weeks) | 35weeks 5 days | C-section | Not Related |
| 30-34 | 88 | Date of LNMP | Premature live birth (< 37 weeks) | 33 weeks 4 days | C-section | Not Related |
| 35-39 | 154 | Date of LNMP | Spontaneous fetal death and/or still birth (> 20 weeks) | 25 weeks | N/A | Not Related |
| 35-39 | 230 | Date of LNMP | Spontaneous fetal death and/or still birth (> 20 weeks) | 41 weeks | Vaginal | Not Related |
| 30-34 | 41 | Clinical examination | Spontaneous fetal death and/or still birth (> 20 weeks) | 13 weeks 6 days | N/A | Not Related |
| 35-39 | 242 | Date of LNMP | Spontaneous fetal death and/or still birth (> 20 weeks) | 40 weeks | Vaginal | Not Related |
| 35-39 | 112 | Early Ultrasound | Spontaneous fetal death and/or still birth (> 20 weeks) | 31 weeks | Vaginal | Not Related |
| 40-44 | 183 | Date of LNMP | Spontaneous fetal death and/or still birth (> 20 weeks) | 30 weeks | Vaginal | Not Related |
| 30-34 | 70 | Early Ultrasound | Spontaneous fetal death and/or still birth (> 20 weeks) | 38 weeks | C-section | Not Related |

LNMP = Last normal menstrual period; C-section = Caesarean section
