# Supplementary material for: Safety, effectiveness and immunogenicity of heterologous mRNA-1273 boost after prime with Ad26.COV2.S among healthcare workers in South Africa: The single-arm, open-label, phase 3 SHERPA study
Source: PLOS Glob Public Health. 2024 Dec 5;4(12):e0003260. doi: 10.1371/journal.pgph.0003260 (PMC11620404; doi:10.1371/journal.pgph.0003260)
Supplement: S1 Fig — Fifty-one samples from HIV-uninfected individuals were tested in both the SARS-CoV-2 lentivirus-based pseudovirus assay and the SARS-CoV-2 VSV-based neutralization assay. Samples were chosen to represent low, middle and high titer values. Titers are depicted as ID50 values for both assays. The correlation between the two assays was measured using the Spearman’s correlation in Graphpad Prism v10.0.2. (DOCX) [file pgph.0003260.s012.docx]

**Supplementary Figure 1: Titer comparison between SARS-CoV-2 lentivirus-based and VSV-based pseudovirus neutralization assays.** Fifty-one samples from HIV-uninfected individuals were tested in both the SARS-CoV-2 lentivirus-based pseudovirus assay and the SARS-CoV-2 VSV-based neutralization assay. Samples were chosen to represent low, middle and high titer values. Titers are depicted as ID50 values for both assays. The correlation between the two assays was measured using the Spearman’s correlation in Graphpad Prism v10.0.2.

**
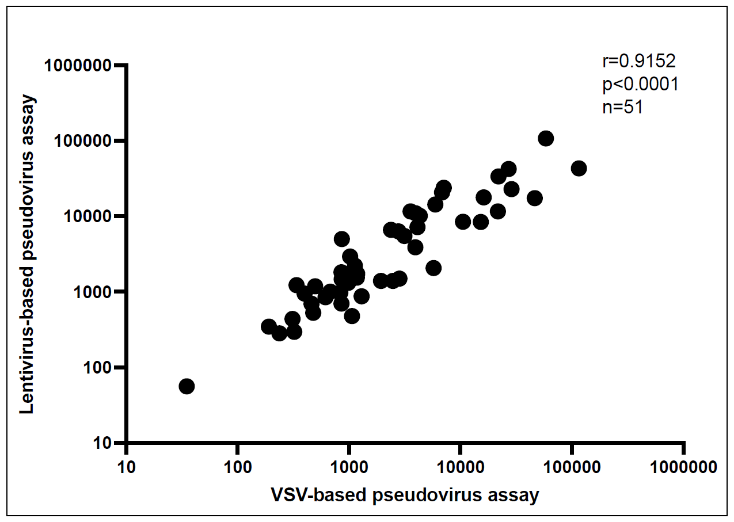
**
