# Supplementary material for: Safety, effectiveness and immunogenicity of heterologous mRNA-1273 boost after prime with Ad26.COV2.S among healthcare workers in South Africa: The single-arm, open-label, phase 3 SHERPA study
Source: PLOS Glob Public Health. 2024 Dec 5;4(12):e0003260. doi: 10.1371/journal.pgph.0003260 (PMC11620404; doi:10.1371/journal.pgph.0003260)
Supplement: S3 Fig — (DOCX) [file pgph.0003260.s014.docx]

**Supplementary Figure 3: Flow diagram showing the adjusted cohort study design**

Adjust for confounders

COVID-19 related hospitalizations/ deaths

No COVID-19 related hospitalizations/ alive

No COVID-19 related hospitalizations/ alive


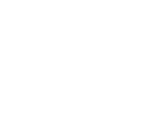


Vaccinated HCWs in Sisonke


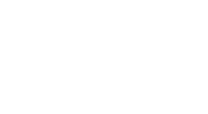


Sisonke Ad26.COV2.S only


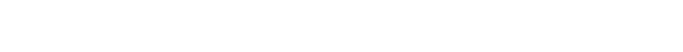


COVID-19 related hospitalizations/ deaths


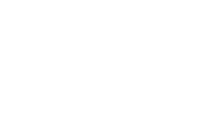


mRNA boosted participants


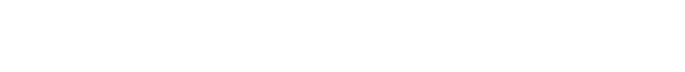

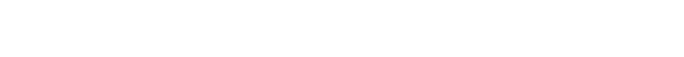

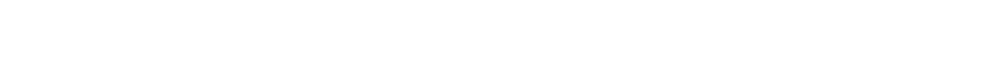


Health care seeking behaviour and selection bias exert an effect. Therefore, **adjusting for potential confounders** is critical
