# Supplementary material for: Safety, effectiveness and immunogenicity of heterologous mRNA-1273 boost after prime with Ad26.COV2.S among healthcare workers in South Africa: The single-arm, open-label, phase 3 SHERPA study
Source: PLOS Glob Public Health. 2024 Dec 5;4(12):e0003260. doi: 10.1371/journal.pgph.0003260 (PMC11620404; doi:10.1371/journal.pgph.0003260)

**Supplementary Figure 4: Comparison of antibody response elicited after mRNA-1273 heterologous boost in HIV-negative participants and PLWH.** The binding (Panel A and D), neutralization (Panel B and C) and ADCC (Panel C and F) were measured in individuals who were HIV-negative and people living with HIV (PLWH). Comparisons were separated based on number of Ad26.COV2.S with 1 doses Ad26.COV2.S (Panels A-C) and 2 doses of Ad26.COV2.S (Panels D-F). The plasma neutralization titer is measured as an ID_50_. Black horizontal bars represent medians. The threshold of detection for the neutralization assay is an ID_50_ of 20. Antibody binding was measured using an in-house SARS-CoV-2 assay using the D614G full spike protein. An EC50 was used to measure the binding titers of the samples. ADCC activity was measured by detecting the crosslinking ability of the antibodies present in the serum. Relative light units were measured which correlate with the levels of FcγRIIIa signalling. For all assays, statistical significance was measured with the Kruskal-Wallis test with Dunn’s multiple comparisons test. Significance is shown as: *p < 0.05, **p < 0.01, ***p < 0.001 and ****p < 0.0001. Medians and fold changes are depicted under each graph. Samples were run in duplicate for all assays.


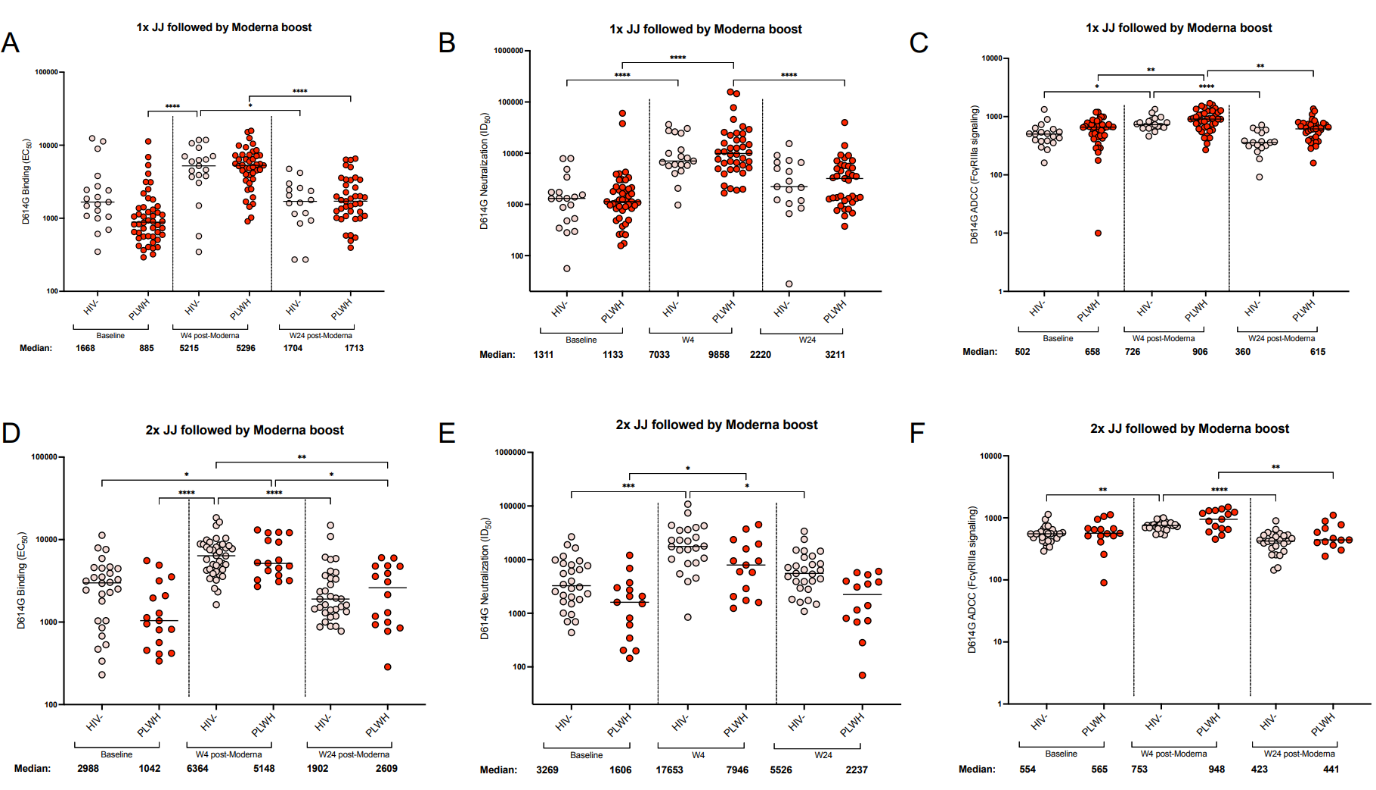

Supplement: S4 Fig — The binding (Panel A and D), neutralization (Panel B and C) and ADCC (Panel C and F) were measured in individuals who were HIV-negative and people living with HIV (PLWH). Comparisons were separated based on number of Ad26.COV2.S with 1 doses Ad26.COV2.S (Panels A-C) and 2 doses of Ad26.COV2.S (Panels D-F). The plasma neutralization titer is measured as an ID50. Black horizontal bars represent medians. The threshold of detection for the neutralization assay is an ID50 of 20. Antibody binding was measured using an in-house SARS-CoV-2 assay using the D614G full spike protein. An EC50 was used to measure the binding titers of the samples. ADCC activity was measured by detecting the crosslinking ability of the antibodies present in the serum. Relative light units were measured which correlate with the levels of FcγRIIIa signalling. For all assays, statistical significance was measured with the Kruskal-Wallis test with Dunn’s multiple comparisons test. Significance is shown as: *p < 0.05, **p < 0.01, ***p < 0.001 and ****p < 0.0001. Medians and fold changes are depicted under each graph. Samples were run in duplicate for all assays. (DOCX) [file pgph.0003260.s015.docx]
