# Supplementary material for: Safety, effectiveness and immunogenicity of heterologous mRNA-1273 boost after prime with Ad26.COV2.S among healthcare workers in South Africa: The single-arm, open-label, phase 3 SHERPA study
Source: PLOS Glob Public Health. 2024 Dec 5;4(12):e0003260. doi: 10.1371/journal.pgph.0003260 (PMC11620404; doi:10.1371/journal.pgph.0003260)

**Supplementary Figure 5: SARS-CoV-2 Infections and circulating Viral strains in South Africa during SHERPA trial 2022-2023 (n=15 392*)**


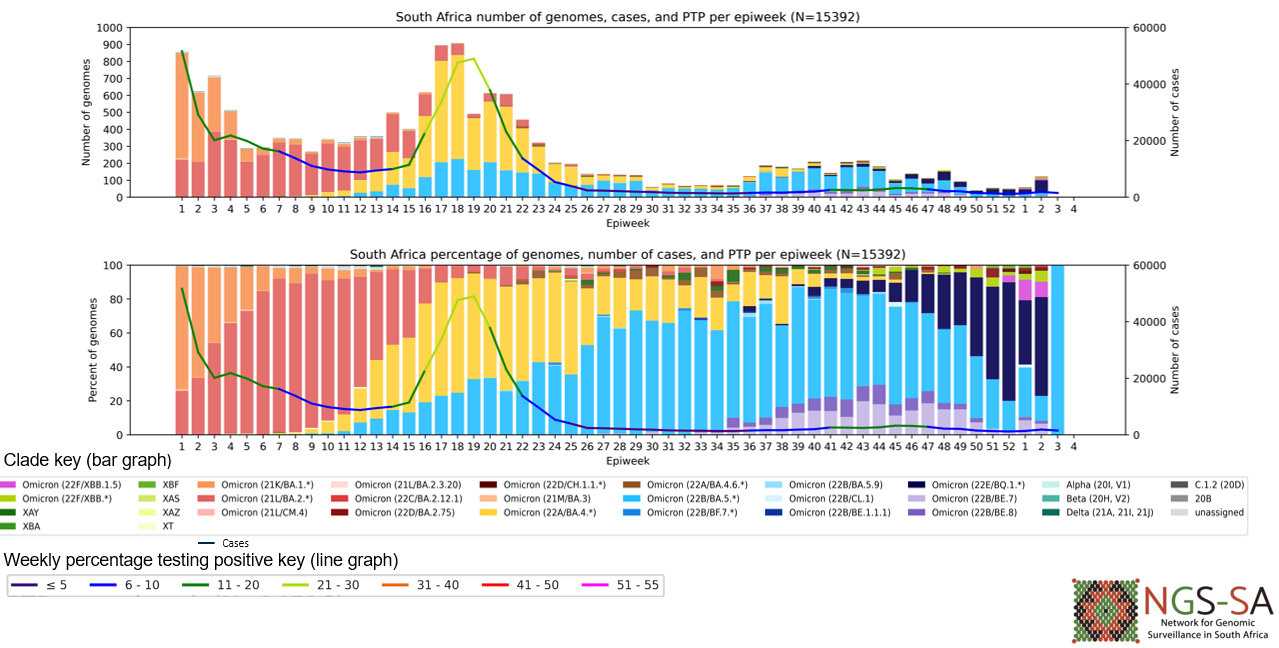

Supplement: S5 Fig — (DOCX) [file pgph.0003260.s016.docx]
