# Supplementary material for: Safety, effectiveness and immunogenicity of heterologous mRNA-1273 boost after prime with Ad26.COV2.S among healthcare workers in South Africa: The single-arm, open-label, phase 3 SHERPA study
Source: PLOS Glob Public Health. 2024 Dec 5;4(12):e0003260. doi: 10.1371/journal.pgph.0003260 (PMC11620404; doi:10.1371/journal.pgph.0003260)
Supplement: S1 Protocol — (PDF) [file pgph.0003260.s017.pdf]

PROTOCOL

Sisonke Heterologous mRNA-1273 boost after prime with Ad26.COV2.S (SHERPA study)

Open-label, phase 3 study to evaluate the effectiveness of heterologous mRNA-1273 boosting of the single or two dose Ad26.COV2.S COVID-19 vaccine among health care workers in South Africa

PROTOCOL Number

Sisonke 4 (SHERPA)/ mRNA-1273-P508

CLINICAL TRIAL SPONSORED BY

South African Medical Research Council (SAMRC)

INVESTIGATIONAL PRODUCTS PROVIDED BY

Moderna

28 February 2022

Version 1.0

I, Glenda Gray have read the Department of Health: *Ethics in health research: principles, processes and structures, second edition*, 2015, the *Guidelines for Good Practice in the Conduct of Clinical Trials with Human Participants in South Africa,* Third Edition, 2020, Department of Health, Pretoria, South Africa (where applicable), and the Declaration of Helsinki (2013) and have prepared this proposal with due cognisance of its content. Furthermore, I will adhere to the principles expressed when conducting this proposed research project.

____

Glenda Gray
28 February 2022

Table of Contents

1. STUDY TEAM 5
1.1 PROTOCOL TEAM 5
1.2 COLLABORATING ORGANIZATIONS 7
1.3 STUDY SITES AND SITE PRINCIPAL INVESTIGATORS 9
2. STUDY SCHEMA 10
3. INTRODUCTION 11
3.1 Study Background 11
3.2 Stakeholder Engagement 13
3.3 Rationale 14
4. mRNA-1273 COVID-19 vaccine 14
4.1 Potential risks of COVID-19 mRNA vaccine 15
4.1.1 Solicited Adverse Reactions 15
4.1.2 Unsolicited Adverse Events 16
4.1.3 Deaths, Other Serious AEs, and Other Significant Unsolicited AEs 16
4.2 Booster Dose Experience 17
4.3 Post-marketing Experience 18
5. Summary of Ad26.COV2.S vaccine data 18
5.1 Safety of Janssen Covid-19 vaccine 19
5.1.1 Unsolicited Adverse Events 19
5.1.2 Immediate Adverse Events 20
5.1.3 Deaths 20
5.1.4 Serious Adverse Events 20
5.1.5 Adverse Events of Special Interest 20
5.2 Clinical safety in post-licensure rollout 21
5.2.1 Clinical safety in the Sisonke Phase 3b implementation study 21
5.3 Rationale for inclusion of Pregnant Women 22
6. OBJECTIVES AND ENDPOINTS 23
7. STUDY DESIGN 24
7.1 Enrolment plan 25
7.2 Study population 25
7.3 Eligibility criteria 25
8. CLINICAL PROCEDURES 26
8.1 Recruitment 27
8.2 Screening and enrolment visit into the main study 27
8.3 Screening and enrolment visit into the safety and immunogenicity sub-study 28
8.4 Follow up visits in the safety and immunogenicity sub-study 28
8.5 Laboratory tests 29
8.6 Administration of study product 29
8.7 Surveillance for hospitalization and breakthrough infections 29
8.8 Surveillance for hospitalization and breakthrough infections 30
9. STUDY PRODUCT 30
9.1 Preparation, Handling and Storage 31
9.2 Study product accountability 31
10. CLINICAL SAFETY 31
10.1 AE reporting 31
10.2 Serious adverse event 32
10.3 Adverse Event of Special Interest (AESI) 33
10.3.1 Anaphylaxis 33
10.3.2 Myocarditis/Pericarditis 33

10.4 Expedited reporting of adverse events 34
10.5 Safety surveillance in the main study 35
10.6 Summary of post vaccination safety monitoring and reporting in the main study 36
10.7 Safety of pregnant women 36
10.8 Safety Oversight 36
10.8.1 Safety Monitoring Committee 37
10.8.2 Protocol Safety Review Team 37
10.8.3 Clinical Safety staff 37
10.9 Safety reporting and review 38
10.9.1 Safety reporting 38
10.9.2 Review of cumulative safety data 38
11. STATISTICAL CONSIDERATIONS 38
11.1 Study design 38
11.2 Statistical analyses 39
11.3 Sample size calculations 40
11.3.1 Adjusted cohort analysis - Primary analysis 40
12. DATA MANAGEMENT 41
12.1 Overview of data management 42
12.2 Data analyses 42
12.3 Data Sharing 42
13. HUMAN SUBJECT PROTECTION AND ETHICAL OBLIGATIONS 43
13.1 Regulatory and Ethical Approval 43
13.2 Informed consent 43
13.3 Risks and Benefits 43
13.4 Monitoring 43
14. REFERENCES 43
15. Appendices 46

1. STUDY TEAM

1.1 PROTOCOL TEAM

**Ameena Goga, MBChB, FC(Paeds), MSc, PhD**<br>**Co-PI**<br>South African Medical Research Council (SAMRC)<br>Francie van Zijl Drive, Parowvallei, Cape Town; 7505 Tygerberg, South Africa<br>E-mail: Ameena.Goga@mrc.ac.za<br>T: +27 12 339 8524	**Nigel Garrett, MBBS, MRCP, MSc, PhD**<br>**Co-PI**<br>Centre for the AIDS Programme of Research in South Africa (CAPRISA)<br>Nelson R Mandela School of Medicine, UKZN, Private Bag X7, Congella 4013, Durban, South Africa<br>Email: nigel.garrett@caprisa.org<br>T: +27 31 655 0617
**Glenda Gray MBChB, FC(Paeds)**<br>**Co-PI**<br>SAMRC**,** Francie van Zijl Drive, Parowvallei, Cape Town; 7505 Tygerberg, South Africa<br>E-mail: glenda.gray@mrc.ac.za<br>T: +27 12 339 8524	**Linda-Gail Bekker, MBChB, DTMH, DCH, FCP(SA), PhD**<br>**Co-PI**<br>The Desmond Tutu HIV Centre<br>University of Cape Town<br>Email: linda-gail.bekker@hiv-research.org.za<br>T: +2721 6506970
**Nonhlanhla Yende-Zuma, MSc, PhD**<br>**Statistician**<br>CAPRISA, Nelson R Mandela School of Medicine, UKZN, Private Bag X7, Congella 4013, Durban, South Africa<br>Email: nonhlanhla.yende@caprisa.org<br>T: +27 31 260 4392	**Tarylee Reddy, MSc, PhD**<br>**Statistician**<br>Biostatistics Research Unit, SAMRC, 491 Peter Mokaba Ridge Road, Overport 4091, Durban, South Africa<br>Email: tarylee.reddy@mrc.ac.za<br>T: +27 31 2034860
**Ian Sanne, MBBCh, DTM&H, FCP(SA), Cert (ID)**<br>**Sub-Investigator**<br>Right to Care/Wits Health Consortium (Clinical HIV Research Unit)<br>Helen Joseph Hospital, Perth Road, Westdene Johannesburg, Gauteng, 2092, South Africa<br>Email: isanne@witshealth.co.za<br>T: +27 11 276 8801	**Azwi Takalani, MBChB, MMed, FCPHM**<br>**Safety Physician**<br>HCRISA<br>Chris Hani Baragwanath Academic Hospital<br>Soweto, 2196 South Africa<br>Email: atakalan@hcrisa.org.za<br>Tel: +27 11 989 9700
**Fatima Mayat, B.Pharm**<br>**Implementation & Oversight Support**<br>Perinatal HIV Research Unit (PHRU)<br>Chris Hani Baragwanath Academic Hospital<br>E-mail: mayatf@phru.co.za<br>T: +27 11 989 9700	**Jackline Odhiambo, B. Pharm, PGDRM, MSc**<br>**Implementation & Oversight Support**<br>HCRISA<br>Chris Hani Baragwanath Academic Hospital<br>Soweto, 2196 South Africa<br>Email: jodhiamb@hcrisa.org.za<br>T: +27 11 989 9700
**Lara Fairall, MBChB, MD**<br>**Sub-Investigator**<br>The Knowledge Translation Unit<br>UCT Lung Institute<br>University of Cape Town<br>Email: Lara.Fairall@uct.ac.za	**Ishen Seocharan**<br>**Data Management**<br>Biostatistics Research Unit, SAMRC, 491 Peter Mokaba Ridge Road, Overport 4091, Durban, South Africa<br>Email: Ishen.Seocharan@mrc.ac.za

**Kubashni Woeber**<br>**Clinical Trial Manager**<br>SAMRC<br>HIV Prevention Research Unit<br>491 Peter Mokaba Ridge | Durban | Kwa Zulu Natal<br>**Email:** Kubashni.Woeber@mrc.ac.za<br>**Tel:** +27 31 242 3730	
**Jonny Peter, MB ChB, MMed, FCP (SA), PhD**<br>**Member of PSRT and advisor**<br>Head of the Division of Allergology and Clinical Immunology at Groote Schuur Hospital, University of Cape Town.<br>Tel: +27 21 442 1871<br>Email: Jonny.Peter@uct.ac.za	**Barry Jacobson, MB ChB, MMed, PhD, FRCS(Glas)**<br>**Member PSRT and advisor**<br>Head of Clinical Haematology, Department of Haematology, NHLS, Johannesburg Hospital and Head of Surgical Research, University of the Witwatersrand, Johannesburg Hospital<br>Tel: +27 11 4898418/5<br>Email: barry.jacobson@wits.ac.za
**Jessica Opie, MBChB Hons (UCT) MRCP (UK) FRCPA (Haem Aus)**<br>**Member of PSRT**<br>Head of Division of Haematology, Department of Pathology, University of Cape Town<br>Tel: +27 21 406 6154<br>Email: Jessica.Opie@uct.ac.za	**Vernon Louw, MBChB (Stell), MMed(Int Med)(Stell), PhD(HPE)(UFS)**<br>**Member of PSRT**<br>Chair and Head of the Division of Clinical Medicine Department of Medicine, University of Cape Town. Tel: +27 21 404 3080<br>Email: Vernon.Louw@uct.ac.za
**Pradeep Rowji, BSc, BSc (Hons), MBBCh (Wits), FCP**<br>**Neurologist**<br>Member of National Executive of the Neurology Association of South Africa<br>Tel: +274805808<br>Email: rowjip@polka.co.za	

1.2 COLLABORATING ORGANIZATIONS

**Collaborating partners**	**Collaborating Organisation**
National Department of Health of South Africa<br>Dr Lesley Bamford<br>National Department of Health<br>Civitas Building, Cnr Thabo Sehume and Struben Streets, Pretoria, 0001<br>Email: Lesley.Bamford@health.gov.za<br>Tel: +27 79 515 8353<br><br>National Institute of Communicable Diseases:<br>Professor Penny Moore:<br>University of the Witwatersrand<br>National Institute for Communicable Diseases, a Division of the NHLS Centre for HIV & STIs: HIV Virology Section, 1 Modderfontein Road, Sandringham, 2131, South Africa<br>Tel: +27 727244501<br>Email: pennym@nicd.ac.za<br><br>Institute of Infectious Disease and Molecular Medicine:<br>Professor Wendy Burgers:<br>University of Cape Town<br>Faculty of Health Sciences, Institute of Infectious Disease and Molecular Medicine, Department of Pathology, Division of Virology: Viral Immunology Unit, Anzio Road, Observatory, 7925, South Africa<br>Tel: +27 825109071<br>Email: wendy.burgers@uct.ac.za<br><br>KwaZulu-Natal Research Innovation and Sequencing Platform (KRISP), Tulio de Oliveira, PhD<br>Sub-Investigator<br>KwaZulu-Natal Research Innovation and Sequencing Platform (KRISP), UKZN, Durban, South Africa<br>Email: deoliveira@ukzn.ac.za<br>T: +27 31 260 4898<br><br>National Institute for Communicable Diseases<br>Harry Moultrie, MBBCh, MMedSc<br>University of the Witwatersrand<br>Johannesburg<br>Phone +27 115475034<br>Email: harrym@nicd.ac.za	**Investigational products provided by:**<br>**Moderna, Inc.**<br>Clinical Lead<br>Brett Leav, MD<br>200 Technology Square<br>Cambridge, Massachusetts USA 02138<br>Email: brett.leav@modernatx.com<br>Phone +16176822724<br><br>Moderna Global Safety Physicians and Pharmacovigilance contact information:<br>Andrea Sutherland<br>Andrea.sutherland@modernatx.com<br><br>Kate Anteyi<br>Kate.antevi@modernatx.com<br><br>Aggregate reports for DSUR<br>aggregatereports@modernatx.com<br><br>Safety Reports to Moderna<br>Drugsafety@modernatx.com<br><br>**Supporting partners**<br>BARC<br>1st Floor Napier House, 11 Napier Road, Richmond, Johannesburg, 2092<br>Phone: +27 11 242 7044<br><br>Biovac<br>Dr Morena Makhoana CEO<br>Tel: +27 21 514 5000<br>Email: Morenam@biovac.co.za<br>Website: www.biovac.co.za<br>15 Alexandra Road, Pinelands, 7405, South Africa<br>Private Bag X3, Pinelands, 7430, South Africa<br><br>Biocair South Africa (Pty) Ltd<br>Unit 18 Pomona Business park<br>57 Maple street<br>Pomona, Kempton Park 1619<br>South Africa<br>Email: southafrica@biocair.com<br>Tel: +27 10 492 3931<br>Leonard N. Lazarus | Africa Regional Director<br>Direct Dial: + 27 10 492 3934 (Extn 2083)<br>Mobile: + 27 83 294 1430

Hutchinson Center Research Institute of South Africa (HCRISA)<br>Chris Hani Baragwanath Academic Hospital<br>Soweto, 2196 South Africa<br>Tel: +27 119899700<br>EMAIL: info@hcrisa.org.za<br><br>Institute of Infectious Disease and Molecular Medicine:<br>Heather Jaspan, MD PhD<br>Health Sciences | University of Cape Town<br>3.37, Falmouth Building, Anzio Road,<br>Observatory 7925<br>+ 27 21 406 6823	Right to Care<br>1006 Lenchen Ave,<br>Centurion Central, Centurion, 0046<br>Tel: +27 11 276 8850<br><br>Clinical Laboratory Services<br>Corner de Korte and Hospital Streets.<br>Braamfontein.<br>Johannesburg. South Africa.<br>Tel: +27 10 001 3900
**Funding for the Sisonke studies is provided by:**<br>National Department of Health<br>South African Medical Research Council (SAMRC)<br>South African National Treasury<br>Moderna	**Study Monitor**<br>Hutchinson Center Research Institute of South Africa<br>Chris Hani Baragwanath Academic Hospital<br>Soweto, 2196 South Africa<br>Email: elleroux@hcrisa.org.za<br>Tel: +27 82 476 8684<br>Tel: +27 60 978 7859<br>Tel: +27 82 659 3740
**ETHICS / REGULATORY OVERSIGHT**	
**UKZN Biomedical Research Ethics Committee**<br>Prof D R Wassenaar<br>Research Office<br>UKZN, Westville, South Africa<br>Email: brec@ukzn.ac.za<br>T: +27 31 260 4495<br>F: +27 31 260 4410<br><br>**UCT Health Science Faculty Research Committee**<br>Prof M Blockman<br>UCT Health Science Faculty<br>Anzio Road<br>Observatory, 7925<br>T: +27 21 650 1236<br>Email: hrec-enquiries@uct.ac.za<br><br>**Sefako Makgatho University Research Ethics Committee (SMUREC)**<br>Prof C Baker<br>Sefako Makgatho Health Sciences University<br>Molotlegi Street, Ga-Rankuwa<br>Pertoria, Gauteng<br>Email: Lorato.phiri@smu.ac.za<br>T: +27 12 521 5617/3044<br><br>**Pharma-Ethics (Pty) Ltd**<br>Dr CSJ Duvenage	**South African Health Products Regulatory Authority (SAHPRA)**<br>Chief Executive Officer:<br>Dr Boitumelo Semete-Makokotlela<br>Email: Boitumelo.Semete@sahpra.org.za<br>T: 012 501 0410/13<br>Chief Regulatory Officer:<br>Portia Nkambule<br>Email: Portia.nkambule@sahpra.org.za<br>C: 078 802 0781 / T: 012 501 0414<br><br>**SAMRC Human Research Ethics Committee**<br>Prof D du Toit<br>Francie Van Zijl Drive, Parowvallei, 7505, Cape Town, South Africa<br>Email: adri.labuschagne@mrc.ac.za<br>T: +27 21 938 0687<br><br>**University of Stellenbosch Health Research Ethics**<br>Prof B Pretorius<br>Health Research Office: Stellenbosch University, Tygerberg Campus, Research Development & Support Division, Room 5008A, Tygerberg Campus Education Building, Francie van Zijl Drive, Tygerberg, 7505<br>E-mail: blanchep@sun.ac.za

123 Amkor Road<br>Lyttelton Manor, 0157, South Africa<br>PO Box 786, Irene, 0062<br>Email: marzelle@pharma-ethics.co.za and colette@pharma-ethics.co.za<br>T: +27 087 230 8460<br>F: +27 12 664 7860	T: +27 21 938 9075<br>**University of Witwatersrand Human Research Ethics Committee (Medical).**<br>Prof C Penny<br>8 Blackwood Avenue<br>Parktown, 2193<br>South Africa<br>Email:<br>T: +27 22 274 9200<br>F: +27 11 274 9281

1.3 STUDY SITES AND SITE PRINCIPAL INVESTIGATORS

City	PI Name	Site Name
Port Elizabeth	Malan, Daniel	PHOENIX Pharma (Pty) Ltd
Cape Town, Groote Schuur	Kassim, Sheetal	Desmond Tutu Health Foundation Clinical Trials Unit
Durban	Naicker, Nivashnee	CAPRISA eThekwini Clinic
Cape Town	Diacon, Andreas	TASK - Central, Eden, Brooklyn Chest Hospital, Delft Day Hospital premises, Dr Ivans Toms Clinic, 1 Smal Street-Bellvile.
Soweto (Chris Hani Baragwanath Hospital)	Lazarus, Erica	Perinatal HIV Research Unit (PHRU) – CH Baragwanath CRS
Worcester	Barnabas, Shaun	FAM-CRU (Family Clinical Research Unit)
Soweto (Kliptown)	Lazarus, Erica	Perinatal HIV Research Unit (PHRU) - Kliptown CRS
Pretoria	Ahmed, Khatija	Setshaba Research Centre
Durban (situated on the grounds of RK Khan hospital)	Naidoo, Logashvari	SA Medical Research Council - Chatsworth
Cape Town, Crossroads/Nyanga	Mahoney, Scott	Desmond Tutu HIV Foundation - Emavundleni Research Centre
Johannesburg (Helen Joseph)	Badal-Faesen, Sharlaa	Themba Lethu HIV Research Unit (CHRU) Clinical HIV Research Unit (CHRU)
Rustenburg	Brumskine, William	The Aurum Institute Rustenburg Clinical Research Centre
Cape Town, Guinea fowl Road Fishhoek	Gill, Katherine	Desmond Tutu HIV Foundation Desmond Tutu Health Foundation (DTHF) Masiphumelele Clinic
Klerksdorp	Innes, Craig	The Aurum Institute Klerksdorp Clinical Research Centre
Ladysmith	Kotze, Philippus	Qhakaza Mbokodo Research Clinic (QM)
Bloemfontein	Lombaard, Johannes	Josha Research
Tembisa	Mngadi, Kathryn	The Aurum Institute Tembisa Clinical Research Centre
Worcester	Luabeya, Angelique	SATVI, Brewelskloof Hospital

Botha's Hill, Kwa-Zulu Natal	Spooner, Elizabeth	SA Medical Research Council - Botha's Hill
Tongaat, KwaZulu Natal	Naicker, Vimla	SA Medical Research Council - Tongaat
Pretoria, Medunsa	Nchabeleng, Maphoshane	MECRU Clinical Research Unit
Mthatha	Dubula, Thozama	Nelson Mandela Academic Clinical Research Unit (NeMACRU)
KwaZulu Natal	Makhaza, Disebo	CAPRISA Vulindlela Clinic
Middelburg	Petrick, Friedrich	Mzansi Ethical Research Centre
Dennilton, Limpopo	Maboa, Rebone Molobane	Ndlovu Research Centre
Pretoria	Grobbelaar, Coert	The Aurum Institute Clinical Research Centre Pretoria

2. STUDY SCHEMA

**Purpose**	The purpose of the study is to evaluate the **effectiveness** of the **heterologous mRNA-1273 (Moderna) boost** against COVID-19 infections and severe disease (including deaths) among health care workers (HCWs) who participated in the Sisonke trial and received either a single dose or two doses of Ad26.COV2.S (Janssen, Johnson and Johnson) COVID-19 vaccine as their primary vaccination. Furthermore, the study aims to collect additional **safety** and **immunogenicity** data on the heterologous mRNA-1273 boost among Sisonke participants.
**Study design**	Open-label, phase 3 COVID-19 mRNA vaccine booster study nested in the larger Sisonke Phase 3b implementation trial population
**Rationale**	South Africa is severely affected by the global COVID-19 pandemic. As part of the **Sisonke study**, HCWs received a single dose of Ad26.COV2.S vaccine between 17 February and 17 May 2021, and were then offered a second dose between 8 November and 17 December 2021. Immunogenicity studies have since indicated that a **heterologous mRNA vaccine boost strategy** may elicit stronger neutralizing antibody responses among people who received the Ad26.COV2.S and other vector-based vaccines.
**Study participants**	Approximate sample size of up to **N=15 000** Sisonke participants of a total target population of N=477 234. Participants will be aged 18 and over who work in the South African public and private health care sector and received either a single dose (N~245 000) or two doses (N~230 000) of Ad26.COV2.S and are willing to receive a mRNA-1273 booster as soon as possible (if single dose of Ad26.COV2.S) or at least 3 months after the second dose (if two doses of Ad26.COV2.S were administered).
**Study sites**	Approximately **30 clinical research sites** that also took part in the Sisonke (VAC31518COV3012) Trial in South Africa with up to approximately **500 participants per site.**

**Study duration**	Participants will receive a heterologous mRNA-1273 boost during an enrolment period of approximately 3 months and will then be followed for **1 year**. We will monitor outcomes by utilising existing surveillance systems including the hospitalisation DATCOV surveillance system, national mortality data and the NHLS/NICD SARS COV-2 testing databases for up to 1 years post initial vaccination. These surveillance systems have already been successfully integrated and utilized as part of the Sisonke study.
**Study products**	mRNA-1273 booster vaccine by Moderna at the 50mcg dose, administered as a single intramuscular injection.
**Primary objectives**	• To compare the effectiveness of Ad26.COV2.S vaccine with a heterologous mRNA-1273 boost on **severe COVID-19** (including hospitalizations and deaths) in Sisonke participants, with Sisonke populations not boosted with mRNA-1273.<br>• To compare the effectiveness of Ad26.COV2.S vaccine with a heterologous mRNA-1273 boost on **any COVID-19 cases**, as compared with Sisonke populations not boosted with mRNA-1273.
**Secondary objectives**	• To compare the effectiveness of a **single dose of Ad26.COV2.S vaccine with or without a heterologous mRNA-1273 boost** on any COVID-19 infection, COVID-19 hospitalizations and deaths in Sisonke participants.<br>• To compare the effectiveness of **two doses of Ad26.COV2.S vaccine with or without a heterologous mRNA-1273 boost** on any COVID-19 infection, COVID-19 hospitalizations and deaths in Sisonke participants.<br>• To compare the effectiveness of a **single dose versus two doses of Ad26.COV2.S vaccine with a heterologous mRNA-1273 boost** on any COVID-19 infection, COVID-19 hospitalizations and deaths in Sisonke participants.<br>• To monitor **safety** among participants, including in pregnant and breastfeeding women, receiving a heterologous mRNA-1273 boost either after a single dose or two doses of Ad26.COV2.S vaccine.<br>• In a subset of participants (approximately N=200), measure the early (Day 29) and late (Month 6) **humoral and cellular immune responses** to heterologous mRNA-1273 boost either after a single dose or two doses of Ad26.COV2.S vaccine.<br>• To monitor the genetic diversity of breakthrough SARS CoV-2 infections.

3. INTRODUCTION

3.1 Study Background

The global COVID-19 pandemic has had a devastating effect on South Africa. As of 02 January 2022, there have been more than 3.4 million recorded cases and 91 198 deaths with more than a quarter of a million excess deaths since May 2020. In addition, dramatic increases in hospitalizations and pressure on the health care system during the third wave, led to excess

deaths estimated to be at least twice as high as those reported previously. The second, third and fourth waves were fuelled by variants of concern (first Beta, then Delta and most recently Omicron), which have increased transmissibility by 2 to 3-fold and had a negative impact on vaccine efficacy.

Nevertheless, South African researchers and policy makers have led the way in contributing to the international COVID-19 response by conducting several clinical vaccine trials and informing the global understanding of the importance of new viral variants by viral genetic surveillance. Variants of concern include the Beta (B.1.351), the Delta (B.1.617.2) and Omicron (B.1.1.529) variants of concern, some of them were first identified in South Africa and are now circulating globally. The Omicron variant virus has been spreading rapidly with increased transmissibility, higher rates of re-infection and evidence of more breakthrough infections in vaccinees.

Recently, an international randomized, double-blind, placebo-controlled phase 3 study (ENSEMBLE trial) that assessed the efficacy and safety of the Ad26.COV2.S COVID-19 (JnJ) vaccine in adults was conducted across four continents (1). Ad26.COV2.S is a monovalent vaccine composed of a recombinant, replication-incompetent adenovirus type 26 (Ad26) vector, constructed to encode the severe acute respiratory syndrome coronavirus-2 (SARS-CoV-2) spike (S) protein. South African trial sites contributed almost 7 000 participants to this trial of approximately 44 000 enrolled participants globally. The study is being conducted under the sponsorship of Janssen (Janssen Vaccines & Prevention B.V) in collaboration with Operation Warp Speed (OWS), which also encompasses the Biomedical Advanced Research and Development Authority (BARDA), the National Institutes of Health (NIH), and the COVID-19 Prevention Trials Network (COVPN) in the US.

The single-dose vaccine candidate demonstrated 66% effectiveness overall in preventing moderate and severe COVID-19 disease, 28 days post-vaccination (72% in the USA; 64% in South Africa). It was 85% effective overall in preventing severe disease, and there were no COVID-19 related hospitalizations and deaths, including in South Africa. Importantly, there was a high level of protection observed against severe disease caused by the Beta variant lineage observed in South Africa (89% as of 28 days after vaccination). These results were important in that they demonstrated vaccine efficacy *in vivo* against the new variant strains. There were no safety issues in this trial and similar vaccines using the same platform have been used in >200 000 people to date.

The Sisonke phase 3B open-label implementation study vaccinated health care workers (HCWs) with the single dose Ad26.COV2.S vaccine during two phases of the South African Covid-19 epidemic, dominated first by the Beta and then by the Delta variant of concern. HCWs were initially vaccinated over 3 months (17 February - 17 May 2021). Vaccine effectiveness (VE) against Covid-19 related hospitalisation, hospitalisation requiring critical or intensive care and death, ascertained 28 days or more post vaccination was assessed up until 17 July 2021 (2). Nested sub-cohorts from two national medical scheme administrators/ managed care organisations comparing unvaccinated population counterparts matched for COVID-19 risk, were evaluated to assess VE using a matched retrospective cohort design. To validate VE we also compared HCWs with matched unvaccinated HCWs in a nested sub-cohort using a provincial health service data system.

Over the 3-month period, 477 234 HCWs were vaccinated in 122 vaccination sites across South Africa. VE derived from the A and B datasets comprising 215 813 HCWs was 83% (95% CI 75-89) to prevent Covid-19 deaths, 75% (95% CI 69-82) to prevent hospital admissions requiring critical or intensive care and 67% (95% CI 62-71) to prevent Covid-19 related hospitalisations. The VEs for all three outcomes were consistent across the three datasets. The VE was maintained in older HCWs and those with comorbidities including those living with HIV. VE remained consistent throughout the Beta and Delta dominant phases of the study.

The single-dose Ad26.COV2.S demonstrated effectiveness against severe COVID-19 disease and death post -vaccination, and against both Beta and Delta variants providing real world evidence for its use globally. The vaccination was also deemed to be safe (3, 4).

Following the results of the ENSEMBLE 2 study, which demonstrated improved vaccine efficacy of a two-dose regimen of Ad26.COV.2 vaccine given 2 months apart, the Sisonke study was expanded to offer a booster dose of the Ad26.COV.2 to all participants. Sisonke 2 enrolled 227 310 HCW (approximately 45% of the Sisonke population) between 8 November and 17 December 2021. Enrolment commenced before the onset of the Omicron driven fourth wave in South Africa providing the opportunity to evaluate early VE in preventing hospital admissions of a homologous boost of the Ad26.COV.2 vaccine given 6-9 months after the initial vaccination in HCW. The Sisonke study team estimated VE of the Ad26.COV2.S vaccine booster in 69 092 HCW as compared to unvaccinated individuals enrolled in the same managed care organization using a test negative analytical design, and compared VE against COVID-19 admission for Omicron during the period 15 November to 20 December 2021. After adjusting for confounders, the study team observed that VE for hospitalisation increased over time since booster dose, from 63% (95%CI 31-81%) to 84% (95% CI 67-92%) and then 85% (95% CI: 54-95%), 0-13 days, 14-27 days, and 1-2 months post-boost. This provided the first evidence of the effectiveness of a second Ad26.COV.2 vaccine dose given 6-9 months after the initial vaccination during a period of omicron variant circulation. This data is important given the increased reliance on the Ad26.COV.2 vaccine in low- and middle-income settings (5).

While these early effectiveness data on two doses of the Ad26.COV.2 vaccine are promising, there has been increasing interest in offering heterologous boosters with mRNA vaccines to people who received a vector based primary regimen. This concept has been implemented at population level in Canada and Europe for the ChimpAd vaccine prime (AstraZeneca) and more recently for the Ad26.COV.2 prime in the US. Systematic reviews show that heterologous (vector based followed by mRNA) vaccinations elicit high neutralizing antibody titres as well as T cell responses (6), almost comparable to homologous mRNA vaccine regimens, and that these regimens are safe. In the Sisonke 2 study, there was a demand by some HCWs to receive a mRNA boost, which could have negatively impacted the uptake for the Ad26.COV.2 boost. As many countries are now offering a third mRNA vaccine booster to their citizens in the face of the Omicron epidemic, the Sisonke study provides a unique opportunity to test heterologous boosting in the South African setting.

This study will utilize the Moderna COVID-19 mRNA vaccine (mRNA-1273) that received an Emergency Use Authorization (EUA) by the US Government's COVID-19 Vaccine Response and is now widely used across the US and Europe. The aim of the study is to compare the effectiveness of the Ad26.COV2.S vaccine (either one or two doses) with a heterologous mRNA-1273 boost on COVID-19 infections, severe COVID-19, hospitalizations and deaths in Sisonke participants, as compared with Sisonke populations who choose not to be boosted with mRNA-1273. Furthermore, the study will monitor the safety of the heterologous mRNA-1273 boost after Ad26.COV2.S vaccination, thereby providing SAHPRA and regulators with urgent safety data on mRNA-1273 in the South African setting. Finally, in a subset of participants (approximately 200 participants) we aim to measure the humoral and cellular immune responses to heterologous mRNA-1273 boosting after either a single dose or two doses of Ad26.COV2.S vaccine.

3.2 Stakeholder Engagement

Recent news and social media activity regarding vaccination and COVID-19 vaccination in general suggest that a great deal of factual, effective and robust engagement in all sectors of society globally are required to optimize access and delivery of COVID-19 vaccines. Although South Africa has had a good profile for vaccination willingness, there has been an increase in vaccine hesitancy and even denialism (the so called 'antivax' ideology) in recent months.

Every effort will be made to support and communicate the study findings and the larger Sisonke communication effort to disseminate accurate information about boosting in Sisonke as part of this nested study. For these reasons, there will be continued public engagement that includes all Sisonke participants.

3.3 Rationale

Since the emergence of the global pandemic of COVID-19, associated with high morbidity and mortality in 2019, two highly efficacious SARS-CoV-2 mRNA vaccines developed against the spike (S) protein of the ancestral strain circulating in Wuhan (Wuhan-Hu-1, D614) have been approved or authorized for emergency use in the United States and other countries, leading to expanded access and widespread use. These are the 30 mcg Pfizer-BioNTech BNT162b2 mRNA vaccine and the 100 mcg Moderna mRNA-1273 vaccine (7,8). In addition, there are a number of other vaccines against SARS-CoV-2 being used worldwide that use other, non-mRNA technologies such as adenovirus vector vaccines, inactivated virus vaccines, and subunit vaccines.

Evidence from clinical trials and real-world effectiveness studies have consistently demonstrated the efficacy of mRNA vaccines in preventing symptomatic disease among adults and children (9-14). More recently, data has shown that a third dose of mRNA vaccines generates good neutralizing antibody and T cell responses against variants of concern, including against Omicron. Therefore, several countries like the US, Israel and European countries have opted to offer their populations mRNA booster doses. As primary vaccination levels are still not high enough in many LMICs, discussions on boosters have been somewhat delayed. Nevertheless, South Africa has decided to move forward with offering homologous boosters as of 2022.

Considering good safety and immunogenicity data from heterologous mRNA boost studies, the Sisonke study provides the ideal opportunity to test mRNA boosting with 50 mcg of the Moderna mRNA-1273 vaccine after a single or two doses of Ad26.COV2.S among HCWs in South Africa and also provide SAHPRA with local data on safety and effectiveness to support licensure. A study schema of the Sisonke population is shown in Table 1.

Table 1: Study schema of Sisonke mRNA-1273 boost and comparator groups

Study Groups	Sisonke (Feb–May 2021)	Sisonke 2 (Nov-Dec 2021)	Sisonke mRNA boost (Mar-May 2022)
mRNA boost Group 1	Ad26.COV2.S	Ad26.COV2.S	mRNA-1273
mRNA boost Group 2	Ad26.COV2.S	X	mRNA-1273
Sisonke Comparator Group 1	Ad26.COV2.S	Ad26.COV2.S	X
Sisonke Comparator Group 2	Ad26.COV2.S	X	X

4. mRNA-1273 COVID-19 vaccine

The mRNA-1273 COVID-19 vaccine developed and manufactured by Moderna, Inc. is a lipid nanoparticle (LNP) dispersion of a messenger ribonucleic acid (mRNA) encoding the prefusion stabilized S protein of SARS-CoV-2 formulated in LNPs composed of 4 lipids (1 proprietary and 3 commercially available). This vaccine has been issued an EUA by the US Food and Drug Administration (FDA) and was granted a conditional marketing authorization (CMA) by the European Commission for active immunization to prevent COVID-19 caused by

SARS-CoV-2 virus in individuals 18 years and older. The vaccine has been granted Emergency Use Listing (EUL) by the World Health Organization but has not been formally licensed by the SAHPRA for active immunization to prevent COVID-19, yet.

The mRNA-1273 vaccine is a suspension for intramuscular injection (IM) administered as a series of two doses (100 mcg in 0.5 mL each) 1 month apart or as a single dose of 50 mcg booster dose. In this study, therefore a booster dose of 50 mcg in 0.25 mL will be administered IM at ≥3 months after the last Ad26.COV2.S dose.

Table 2: Summary of clinical studies in adults establishing safety, immunogenicity, and vaccine efficacy in the general population

ClinicalTrials.gov Identifier	Study number	Phase	N	Dose groups	Route	Schedule	Reference
NCT04283461	P101	1	120	25, 50, 100, or 250 mcg	IM	M0, M1	(90, 91)
NCT04405076	P201	2	600	50 or 100 mcg	IM	M0, M1	(92-94)
NCT04470427	P301	3	30,351	100 mcg	IM	M0, M1	(92-94)

On December 18, 2020, after review of the available safety and efficacy data from an ongoing phase 3 trial (P301), the FDA issued an EUA for emergency use of Moderna COVID-19 Vaccine (mRNA-1273) for the prevention of COVID-19 for individuals 18 years of age and older. As of September 2021, over 145 million doses of the mRNA-1273 vaccine and 210 million doses of the Pfizer-BioNTech mRNA vaccine had been administered to adults in the US without any serious reactogenicity under the VAERS reporting system and v-safe smart-phone tool for reporting side effects after getting a COVID-19 vaccine (15). Approximately 55 million doses of the Moderna COVID-19 vaccine and 400 million doses of the Pfizer-BioNTech BNT162b2 vaccine had been administered under the CMA in European countries. The FDA granted approval for the Pfizer-BioNTech vaccine Biological License Application on 23 August 2021 and approval of the Moderna mRNA-1273 vaccine (marketed as Spikevax (COVID-19 Vaccine, mRNA) on 31 January 2022.

4.1 Potential risks of COVID-19 mRNA vaccine

4.1.1 Solicited Adverse Reactions

The safety profile presented below is based on data generated in an ongoing placebo-controlled clinical study on 30 346 participants ≥ 18 years of age. Solicited adverse reactions were reported more frequently among vaccine participants than placebo participants. The most frequently reported adverse reactions after any dose in the vaccine group were pain at the injection site (92.0% any grade; 6.1% grade ≥ 3), fatigue (70.1% any grade; 10.1% grade ≥ 3), headache (64.9% any grade; 5.8% grade ≥ 3), myalgia (61.6% any grade; 9.1% grade ≥ 3), arthralgia (46.5%; 5.4% grade ≥ 3), and chills (45.5% any grade; 1.4% grade ≥ 3). The majority of local and systemic adverse reactions had a median duration of 1 to 3 days. Overall, there was a higher reported rate of adverse reactions in adults aged 18 to < 65 years than in those aged 65 years and above.

Grade 3 solicited local adverse reactions were more frequently reported after Dose 2 than after Dose 1. In participants who received the vaccine, solicited systemic adverse reactions were reported more frequently after Dose 2 than after Dose 1. Grade 3 systemic adverse reactions were reported more frequently after Dose 2 than after Dose 1.

4.1.2 Unsolicited Adverse Events

Summaries of unsolicited adverse events (AEs) are available for the 28-day post-injection follow-up period and with accumulated safety data through a median duration of approximately 6 months after the second injection.

The incidence of treatment-emergent adverse events (TEAEs) was similar between the mRNA-1273 and placebo treatment groups in the 28-day follow-up period (31.3% and 28.6%, respectively) and in the overall follow-up period (41.6% and 43.0%, respectively). The commonest reported unsolicited TEAEs in the mRNA-1273 and placebo groups, respectively, were fatigue (5.0% vs. 4.4%) and headache (4.9% vs. 4.5%) during the 28-day follow-up period and during the follow-up period (fatigue [5.8% vs. 5.3%] and headache [6.0% vs. 5.8%]).

Commonly reported AEs were generally consistent with the solicited adverse reactions captured to assess reactogenicity. During the 28-day follow-up period, unsolicited TEAEs that were reported for ≥ 1% of participants in the mRNA-1273 group and showed higher incidence compared with placebo were lymphadenopathy (1.7% vs. 0.8%), injection site pain (1.7% vs. 0.8%), and injection site erythema (1.0% vs. 0.3%). Other TEAEs that were reported for ≥ 1% of participants in the mRNA-1273 group and did not show higher incidence compared with the placebo group were arthralgia, myalgia, diarrhea, cough, nausea, oropharyngeal pain, nasal congestion, and hypertension (arthralgia, myalgia, and nausea were solicited systemic ARs and were only reported as TEAEs if they started on Day 8, or persisted after Day 7, after the most recent injection). Observations were similar with follow-up for the duration of the overall follow-up period.

In the mRNA-1273 group, the incidence of local adverse reactions with delayed onset (reaction started on Day 8 or afterwards) was higher after the first injection (80 participants [0.5%]) than after the second injection (10 participants [< 0.1%]). The most common adverse reaction that was first reported on Day 8 or later was erythema, which was reported for 68 participants (0.4%) in the mRNA-1273 group after the first injection and 6 additional participants (< 0.1%) in the mRNA-1273 group after the second injection.

Hypersensitivity AEs were reported in 2.2% of vaccine recipients and 1.8% of placebo recipients. Hypersensitivity events in the vaccine group included injection site rash and injection site urticaria, which are likely related to vaccination. There have been no cases of severe hypersensitivity or anaphylactic reactions reported immediately after vaccination in the trial to date.

In the 28-day period, facial paralysis was reported for 2 participants in the mRNA-1273 group and 1 participant in the placebo group. During the overall follow up period, facial paralysis was reported for 8 participants in the mRNA-1273 group and 3 participants in the placebo group.

4.1.3 Deaths, Other Serious AEs, and Other Significant Unsolicited AEs

A total of 32 deaths were reported in the overall follow up period, including 16 participants (0.1%) in each group. No trends were apparent in the timing or causes of deaths. Baseline age was ≥ 65 years for 9 of the participants in the mRNA-1273 group and 6 participants in the placebo group. None of the unsolicited TEAEs leading to death were considered to be related to the investigational product (IP).

No difference was observed between the groups in the rates of reported SAEs during Part A (1.8% in the mRNA-1273 group [401 events] and 1.9% in the placebo group [439 events]). COVID-19 was reported as a serious TEAE for 2 (< 0.1%) participants in the mRNA-1273 group and for 40 (0.3%) participants in the placebo group. No other event was reported as an SAE in ≥ 0.1% of participants in either group. At least 1 SAE that was considered related to treatment was reported for 12 participants (< 0.1%) in the mRNA-1273 group and 4

participants (< 0.1%) in the placebo group. Swelling of the face was reported as a treatment-related SAE for 2 participants in the mRNA-1273 group and 1 participant in the placebo group; no other event was reported as treatment-related for more than 1 participant in either group or overall.

The incidence of SAEs within the 28-day follow-up period was lower in both groups than in the overall follow up period and similarly balanced (0.6% in the mRNA-1273 group [140 events] and 0.7% in the placebo group [147 events]). The participant incidence of unsolicited TEAEs leading to discontinuation from the study vaccine in the overall follow up period was lower in the mRNA-1273 group (0.5%; 87 events) than in the placebo group (0.7%; 123 events). The most frequently reported TEAE leading to discontinuation from the study vaccine was COVID-19 (14 participants [< 0.1%] in the mRNA-1273 group vs. 53 participants [0.3%] in the placebo group). Urticaria was reported by 5 participants who received mRNA-1273 and 2 participants who received placebo (< 0.1% each); no other unsolicited TEAE (preferred term [PT]) leading to discontinuation from the study vaccine was reported by more than 3 participants in either group.

The participant incidence of unsolicited TEAEs leading to discontinuation from participation in the study up to 28 days after any injection was similar between the mRNA-1273 group (< 0.1%; 9 events) and the placebo group (< 0.1%; 6 events). No unsolicited TEAE (PT) leading to discontinuation from participation in the study up to 28 days after any injection was reported by more than 1 participant in either group.

The participant incidence of unsolicited TEAEs leading to discontinuation from participation in the overall follow up period was similar between the mRNA-1273 group (0.2%; 26 events) and the placebo group (0.2%; 23 events). Myocardial infarction was reported by 2 participants in the mRNA-1273 group and 4 participants in the placebo group, and COVID-19 was reported in 1 participant in the mRNA-1273 group and 3 participants in the placebo group (< 0.1% each); no other unsolicited TEAE (PT) leading to discontinuation from participation in the study was reported by more than 2 participants in either group.

4.2 Booster Dose Experience

In a Phase 2, randomized, observer-blind, placebo-controlled, dose-confirmation study to evaluate the safety, reactogenicity, and immunogenicity of the mRNA-1273 vaccine in participants 18 years of age and older, 198 participants received 2 doses (0.5 mL 1 month apart) of the mRNA-1273 vaccine primary series. In an open-label phase of this study, 167 of those participants received a single booster dose (0.25 mL) at least 6 months after receiving the second dose of the primary series. The solicited adverse reaction profile for the booster dose was similar to that after the second dose in the primary series.

The safety of the mRNA-1273 vaccine (0.25 mL) booster dose in individuals who completed primary vaccination with another authorized or approved COVID-19 vaccine (heterologous booster dose) is inferred from the safety of the mRNA-1273 vaccine (0.25 mL) booster dose administered following completion of the mRNA-1273 vaccine primary series (homologous booster dose) and from data from an independent Phase 1/2 open-label clinical trial (NCT04889209) conducted in the US that evaluated a heterologous booster dose (0.5 mL) of the Sponsor's COVID-19 vaccine. In this study, adults who had completed primary vaccination with the mRNA-1273 vaccine 2-dose series (N=151), a Janssen COVID-19 Vaccine single dose (N=156), or a Pfizer-BioNTech COVID-19 Vaccine 2-dose series (N=151) at least 12 weeks prior to enrollment and who reported no history of SARS-CoV-2 infection were randomized 1:1:1 to receive a booster dose of one of three vaccines: the mRNA-1273 (0.5 mL), Janssen COVID-19 Vaccine, or Pfizer-BioNTech COVID-19 Vaccine. Adverse events were assessed through 28 days after the booster dose. An overall review of adverse reactions reported following the mRNA-1273 heterologous booster dose (0.5 mL) did not identify any

new safety concerns, as compared with adverse reactions reported following the mRNA-1273 vaccine primary series doses or homologous booster dose (0.25 mL).

4.3 Post-marketing Experience

In the post-authorization period, there have been very rare reports of anaphylaxis following mRNA-1273 administration. In addition, there have been very rare reports of myocarditis and pericarditis occurring after vaccination with COVID-19 mRNA vaccines. The majority of the cases have been reported in young males shortly after the second dose of the vaccine. The risk is highest in males under the age of 40 years, specifically males between 12 to 17 years. Symptoms include chest pain, shortness of breath, or palpitations. Study participants should seek medical attention and notify study site staff if any of these symptoms occur following vaccination. While some cases required intensive care support, available data from short-term follow up suggest that most cases had resolution of symptoms with conservative management. Information is not yet available about potential long-term sequelae. It is not known whether the risk of myocarditis or pericarditis is increased following additional doses of mRNA vaccine. Investigators and study participants should be alert to the signs and symptoms of myocarditis and pericarditis.

Solicited local and systemic reactions to mRNA-based COVID-19 vaccines (Moderna and Pfizer-BioNTech) collected by CDC via the v-safe active surveillance system from more than 3 million people (from December 14, 2020 to February 28, 2021) have recently been published and continue to support a very good safety profile of this vaccine platform (16). Cases of transient, mild myocarditis/pericarditis have been observed largely in young men and more commonly after the 2nd dose, and these events have been exceedingly rare; the CDC has estimated an approximate frequency of 12.5 cases per million doses of second-dose mRNA vaccine among individuals 12-39 years of age (17, 18). Natural SARS-CoV-2 infection has a 5- to 20-fold higher rate of myocardial disease than vaccination with mRNA vaccines (19). Having said this, the CDC has analyzed the risk-benefit of mRNA vaccination for both Moderna and Pfizer-BioNTech and recommended vaccination in all population groups being enrolled in this trial. In addition, clinical trial and real-world data on the safety of a third dose of the Pfizer-BioNTech vaccine has been very reassuring, indicating reactions comparable to those seen after a second dose (16, 20).

5. Summary of Ad26.COV2.S vaccine data

There is considerable clinical trial and real-world effectiveness data that the Ad26.COV2.S administered at 1 or at 2 doses as a primary vaccination strategy is safe and effective at preventing severe COVID-19 infection. Based on recent data, administration of a second JnJ dose resulted in increased protection against symptomatic COVID-19, increased strength and breadth of immune responses against variants of concern and increase protection against severe/critical COVID-19. It substantially increased protection, especially against symptomatic COVID-19, including when caused by SARS-CoV-2 variants of concern. The primary analysis results of Janssen's 2-dose efficacy study COV3009 included data from 7484 participants who received 2 doses of Ad26.COV2.S and 7008 participants who received 2 doses of placebo in the per protocol set. Median follow-up time after the second dose in the double-blind phase was 36 days (0-172 days), with 29.3% of participants in the per protocol set with at least 2 months of follow-up after the 2nd dose. The data is summarized in Table 3 demonstrating a VE of 75% globally against symptomatic COVID-19 and 100% efficacy against severe/critical COVID-19.

Study COV3009 indicates that an Ad26.COV2.S second dose 2 months after the initial dose substantially increases efficacy, especially against symptomatic infections, including when caused by SARS-CoV-2 variants of concern. In the U.S. the boosted population has a vaccine efficacy of 94%, whereas the single dose vaccine efficacy is 70%. Consistent with the efficacy

results, immunogenicity data indicate that a second dose 2 months after the initial dose substantially increases humoral immune responses (ELISA titers) by about 4-fold.

Table 3: Vaccine Efficacy of Two Doses of Ad26.COV2.S

	**COV3009 primary analysis, VE 14 days after Ad26.COV2.S booster dose**	**COV3001 final analysis double-blind phase, VE 28 days after single Ad26.COV2.S dose**
**Symptomatic COVID-19***		
**US**	94% (95% CI 59;100)	70% (95% CI 61;77)
**Global**	75% (95% CI 55;87)	53% (95% CI 47,58)
**Severe/Critical COVID-19**		
**Global**	100% (adjusted 95% CI 33 100.00)	75% (adjusted 95% CI 65;82)

Administration of an Ad26.COV2.S booster dose 6 months after the initial dose further increased humoral immune responses, increasing Ab titers by about 9-12 fold relative to day 29 levels. Therefore, VE against symptomatic infection may increase further. The Ad26.COV2.S vaccine when given as a booster had an acceptable safety profile with no new safety signals. In addition, the following data are provided as supportive information: immunogenicity results of 2-dose vaccination with Ad26.COV2.S ($5x10^{10}$ vp) against variants of concern, results on the impact of neutralizing antibodies against the Ad26 vector on Ad26.COV2.S immunogenicity, and results on the correlation between neutralizing, binding, and functional antibody responses.

5.1 Safety of Janssen Covid-19 vaccine

The most extensive safety information for 2 doses of Ad26.COV2.S at $5x10^{10}$ vp is available from the Phase 3 study COV3009. This study included 31,300 participants, of whom 8,655 participants received a booster dose of Ad26.COV2.S in the double-blind phase. The results of a safety analysis for the double-blind phase are presented below. At the data cutoff for this analysis, 71.2% and 28.4% of participants had completed 2 months of follow-up after the first and booster vaccinations, respectively. Summaries of solicited and unsolicited AEs are based on the Safety Subset, which included 6,068 participants (3,016 in the Ad26.COV2.S group and 3,052 in the placebo group). Summaries of deaths, SAEs, MAAEs, AESIs, and AEIs are based on the full analysis set (31,300 participants; 15,708 in the Ad26.COV2.S group and 15,592 in the placebo group). Overall, 76.4% of participants were white and 52.6% of participants were male. The median age was 53 years (range: 18; 99 years) and 35.9% of participants were ≥60 years of age.

5.1.1 Unsolicited Adverse Events

Overall, unsolicited AEs were reported for 18.6% of participants in the Ad26.COV2.S group and 13.7% of participants in the placebo group. In the Ad26.COV2.S group, unsolicited AEs were reported for 15.1% and 10.2% of participants post-dose 1 and post-booster, respectively. All unsolicited AEs had a frequency <5% by preferred term. The most frequently reported unsolicited AEs post-booster, which were also recorded as solicited AEs, were headache (2.2%), fatigue (1.9%), and myalgia (1.4%). The most frequently reported unsolicited AEs post-booster that were not recorded as solicited AEs were chills (0.5%), oropharyngeal pain and arthralgia (both 0.4%). Most unsolicited AEs were Grade 1 or Grade 2 in severity. Unsolicited AEs of at least Grade 3 in severity were reported for 0.7% and 0.8% of participants in the Ad26.COV2.S group post-dose 1 and post-booster, respectively. Post-dose 1, the most frequently reported unsolicited AE of at least Grade 3 in severity was headache (0.3%). Post-booster, nausea (2 participants [0.1%]) was the only unsolicited AE of at least Grade 3 in severity reported for more than 1 participant. Unsolicited AEs related to vaccination were

reported for 9.4% and 5.1% of participants in the Ad26.COV2.S group post-dose 1 and post-booster, respectively, with the most frequently reported being fatigue (2.7% and 1.4%) and headache (2.6% and 1.4%).

5.1.2 Immediate Adverse Events

The first 1,000 participants remained under observation at the study site for at least 30 minutes after each vaccination to monitor for the development of acute reactions. No early onset had been observed in either age group at the time of the Day 3 safety review of the initial 1,000 participants; therefore, the observation period at the study site could be reduced to at least 15 minutes for the remaining participants based on local country recommendations. Solicited and unsolicited immediate AEs were infrequent (<0.5% of participants post-dose 1 or post-booster). Immediate hypersensitivity reactions following vaccination were rare and nonserious. No immediate severe allergic (anaphylaxis) reactions were reported. Anxiety-related reactions to vaccination, including vasovagal reactions such as syncope and presyncope, were rare (<0.1%), and evenly distributed between the Ad26.COV2.S and placebo groups post-dose 1 and post-booster.

5.1.3 Deaths

Up to the cutoff date of 25 June 2021, 17 deaths were reported during the double-blind phase: 4 in the Ad26.COV2.S group and 13 in the placebo group. Of the 4 deaths reported in the Ad26.COV2.S group, none had a SARS-CoV-2 positive test during the study. The causes of death by preferred term were lung adenocarcinoma and death of unknown cause after the first dose, and cerebral hemorrhage and myocardial infarction after the booster, all of which were considered not related to vaccination. In the placebo group, 6 of the 13 deaths had a positive SARS-CoV-2 test during the study, and the causes of death in these participants were COVID-19 or COVID-19 pneumonia.

5.1.4 Serious Adverse Events

In the double-blind phase of the study, SAEs were reported for 240 participants in the full analysis set (104[0.7%] participants in the Ad26.COV2.S group and 136 [0.9%] participants in the placebo group). A total of 98 (0.6%) participants reported SAEs not associated with COVID-19 in the Ad26.COV2.S group compared with 104 (0.7%) participants in the placebo group. A total of 8 (0.1%) participants reported SAEs associated with COVID-19 in the Ad26.COV2.S group compared with 36 (0.2%) participants in the placebo group. Related SAEs were reported in 8 participants in the Ad26.COV2.S group and 3 participants in the placebo group. In the Ad26.COV2.S group after the first dose, the related SAEs were pyrexia, pericarditis, allergy to vaccine, and hemoptysis in 1 participant each, and injection site swelling, vertigo, and myocardial necrosis marker increased in 1 participant. Related SAEs after the booster dose were facial paresis, pulmonary embolism, and cerebrovascular accident in 1 participant each.

5.1.5 Adverse Events of Special Interest

Following the identification of a safety signal for very rare events of thrombosis with thrombocytopenia syndrome (TTS) in post-marketing data, TTS was considered an AESI in clinical studies. A thrombotic event or thrombocytopenia (defined as platelet count below 150,000/µL) alone was considered a suspected AESI for further investigation. In the double-blind phase of the COV3009, at least 1 suspected AESI (thrombotic event or thrombocytopenia) was reported for 18 (0.1%) participants in the Ad26.COV2.S group (13 participants after the first dose and 5 participants after the booster) and 22 (0.1%) participants in the placebo group. The majority were thromboembolic events, reported for 14 (0.1%) participants in the Ad26.COV2.S group and 18 (0.1%) participants in the placebo group. Thrombocytopenia was reported as a suspected AESI for 4 (<0.1%) participants in the Ad26.COV2.S group and 5 (<0.1%) participants in the placebo group.

Cases for which a thromboembolic event was reported in combination with thrombocytopenia were adjudicated by an internal AESI adjudication committee. No case in the Ad26.COV2.S group met the Brighton Collaboration criteria Level 1 or CDC criteria Tier 1. During the double-blind phase, no cases of thromboembolic events in combination with thrombocytopenia were reported in the Ad26.COV2.S group. Deep vein thrombosis in combination with thrombocytopenia was reported for 1 participant in the Ad26.COV2.S group 100 days post-vaccination. This participant was unblinded before the event and is therefore counted in the open-label phase. The case was assessed as Brighton Collaboration criteria Level 3 and did not meet CDC Tier 1/ 2 criteria based on available data. In the placebo group, 1 participant had deep vein thrombosis on Day 27 (double-blind phase) and subsequently pulmonary embolism on Day 29 (open-label phase) in combination with thrombocytopenia; this case was assessed as Brighton Collaboration Level 1 and did not meet CDC Tier 1/ 2 criteria based on available data.

In COV3009, hemorrhagic disorders were reported for a low percentage of participants (0.4% and 0.2% in the Ad26.COV2.S group and placebo group, respectively). A numerical imbalance in hemorrhagic disorders was observed between the Ad26.COV2.S group and placebo group (55 v 29 in the double-blind phase, 24 v 14 cases in the 28 days post-dose 1, and 17 v 7 cases in the 28 days post-booster). This imbalance was not observed in COV3001 for the primary analysis (22 v 25) or final analysis (48 v 77) of the double-blind phase. In COV3009, 8 hemorrhagic disorders were SAEs. This included 6 SAEs in the Ad26.COV2.S group: cerebral haemorrhage, worsening of haemorrhagic ovarian cyst, haemothorax, upper gastrointestinal bleed and urethral bleeding, which were considered not related to vaccination, and a related event of hemoptysis. In the placebo group, SAEs of gastrointestinal haemorrhage and lower gastrointestinal bleed were reported.

5.2 Clinical safety in post-licensure rollout

On the 13 April 2021 the US based Food and Drug Administration and the Centre for Disease Control issued a recommendation to pause the national roll out of the Janssen Covid-19 vaccine. It was stressed in a briefing that the pause was recommended out of an abundance of caution. While it was reiterated that there have only been 6 cases out of nearly 7 million doses of Janssen Covid-19 vaccine administered, the individualized treatment approach required and the similarities to the AstraZeneca COVID19 vaccine (Vaxevria) adverse events previously reported led to the decision to request a pause. All 6 cases occurred in young women under the age of 50 years. This prompted a similar cautionary pause in the Sisonke 3b study as recommended by human research ethics committees at UCT and UKZN and the SAHPRA and announced publicly by the Minister of Health. The Sisonke leadership initiated a study pause on 13 April 2021. This was done to allow further collection of information on the 6 reported cases and to create and submit an amended Sisonke protocol to SAHPRA and the related human research ethics committees.

5.2.1 Clinical safety in the Sisonke Phase 3b implementation study

The Sisonke study confirmed a good safety profile associated with the Janssen COVID-19 vaccine. Early Sisonke safety results were published in April 2021 (3). Bi-weekly reports have been submitted to SAHPRA and ethics committees and full study safety report has since been published and are summarized here (3,4).

We monitored AEs through self-reporting triggered by text messages after vaccination, through health care provider reports and by active case finding. COVID-19 breakthrough infections, hospitalisations and deaths were ascertained via linkage of the electronic vaccination register with existing national databases. The frequency and incidence rate of non-serious and serious AEs were evaluated until 28 days after the final vaccination on 15 June 2021. Enrolment began 17 February 2021. Of 477,234 participants enrolled, there were 10,279 (2.2%) AE reports of which 139 (1.4%) were serious. Women, who constituted almost

75% of the study population, reported AEs more frequently than men (2.3% vs. 1.6%), AE reports decreased with increasing age (3.2% for 18–30-year-old versus 1.5% in greater 55-year-olds), and participants with previous COVID-19 infection reported slightly more AEs (2.6% vs. 2.1%). The commonest reactogenicity events were headache and body aches, followed by injection site pain and fever, and most occurred within 48 hours of vaccination. Two cases of Thrombosis with Thrombocytopenia Syndrome and three cases of Guillain-Barre Syndrome were reported post-vaccination. Serious AEs and AEs of special interest including vascular and nervous system events, immune system disorders and deaths occurred at lower rates than the expected population rate.

5.3 Rationale for inclusion of Pregnant Women

There is an increased risk of severe COVID-19 disease during pregnancy, as well as an increased risk of adverse birth outcomes. An ongoing systematic review (21) details an analysis into the impact of COVID-19 on pregnant women and their babies. The review found that one in 10 pregnant and recently pregnant women attending or admitted to hospital for any reason were diagnosed with confirmed COVID-19. Overall, 339 pregnant women with confirmed COVID-19 died from any cause (0.02% of a total 41,664 women involved in 59 studies). In this review, the overall rates of stillbirth and neonatal death were low in women with suspected or confirmed COVID-19.

The most common clinical symptoms of COVID-19 in pregnant women were fever (40%) and cough (41%), although compared to non-pregnant women of reproductive age, pregnant and recently pregnant women with COVID-19 were more likely to be asymptomatic. Increased maternal age, high body mass index, non-white ethnicity, and pre-existing comorbidity including chronic hypertension and diabetes were identified as risk factors for pregnant women developing severe COVID-19. While there is emerging evidence from the review that pregnancy specific conditions such as pre-eclampsia and gestational diabetes may be associated with severe covid-19, more data are needed to robustly assess the association between pregnancy specific risk factors and COVID-19 related outcomes.

These data confirm that pregnant women should be considered a high-risk group, particularly those identified to have risk factors for severe COVID-19. Thus, pregnant women were included in the Sisonke sub-study, and are part of national COVID-19 vaccination roll-out programmes globally.

Combined developmental and reproductive toxicity studies in rabbits have been reviewed by the FDA, who concluded that Ad26.COV.S given prior to mating and during gestation periods at doses of 1 $X10^{11}$ VP (2 times the human dose) did not have any adverse effects on female reproduction, fetal/embryonal development or postnatal development, which further justifies the inclusion of pregnant women in COVID-19 vaccine studies.

A developmental toxicity study was performed in female rats administered the equivalent of a single human dose of SPIKEVAX twice prior to mating and twice during gestation. The study revealed no evidence of harm to the fetus due to the vaccine.

The American College of Obstetricians and Gynecologists' Immunization, Infectious Disease and public Health Preparedness Expert Work Group, the USA Center for Disease Control and the World Health Organization have recommended the Janssen and Moderna Covid-19 vaccines for use in pregnant and lactating women. They concluded that pregnant women and lactating women in the USA can receive the mRNA-1273 vaccine. Replication incompetent or replication defective virus vaccines are not contra-indicated in pregnancy. The same type of vaccine has been authorized for use in Ebola and has been studied extensively for other illnesses.

A recent publication of real-world surveillance data in pregnant women receiving the mRNA vaccines has reported overall good safety. A total of 35,691 v-safe participants 16 to 54 years of age identified as pregnant. Injection-site pain was reported more frequently among pregnant persons than among nonpregnant women, whereas headache, myalgia, chills, and fever were reported less frequently. Among 3958 participants enrolled in the v-safe pregnancy registry, 827 had a completed pregnancy, of which 115 (13.9%) resulted in a pregnancy loss and 712 (86.1%) resulted in a live birth (mostly among participants with vaccination in the third trimester). Adverse neonatal outcomes included preterm birth (in 9.4%) and small size for gestational age (in 3.2%); no neonatal deaths were reported. Although not directly comparable, calculated proportions of adverse pregnancy and neonatal outcomes in persons vaccinated against Covid-19 who had a completed pregnancy were similar to incidences reported in studies involving pregnant women that were conducted before the Covid-19 pandemic. Among 221 pregnancy-related adverse events reported to the VAERS, the most frequently reported event was spontaneous abortion (46 cases) (22). In addition, another analysis reported that the odds of having received a recent COVID-19 vaccine were not higher among women with spontaneous abortions than for women with ongoing pregnancies (23).

We acknowledge that safety data for COVID-19 vaccines in pregnancy and breastfeeding are still accumulating. International and local experts and groups such as the College of Obstetricians and Gynaecologists of South Africa, the World Health Organization, the International Federation of Obstetrics and Gynaecologists, the US CDC, the United Kingdom's Joint Committee on Vaccination and Immunisation, the American College of Obstetrics and Gynaecology and the Royal College of Obstetricians and Gynaecologists have remained united in strongly recommending vaccination for pregnant and breastfeeding women given large amounts of safety data for similar ''non-live' vaccines. Pregnancy exposure safety data for >100,000 exposed pregnancies for other 'non-live' vaccines including seasonal influenza and tetanus, diphtheria, pertussis and poliomyelitis polyvalent vaccines show no safety concerns. Animal studies for mRNA and the Janssen Covid-19 vaccine showed no concerns, and the Ebola vaccine, which uses the same adenovirus vector as the Janssen Covid-19 vaccine, has been used widely including pregnant women with no concerns. It is therefore considered highly unlikely that such vaccines would be harmful if administered in pregnancy or breastfeeding.

In this study, pregnant and breastfeeding women will be included. Safety of the heterologous mRNA-1273 booster will be monitored making use of the larger Sisonke study safety monitoring systems to evaluate AEs and pregnancy outcomes (including live births, live preterm birth, still born or abortions). We will also evaluate the number of participants with pregnancy related AEs, and the number of pregnant women with AEs or SAEs compared with the general population. In addition, pregnant participants or breastfeeding participants will be included in the safety and immunogenicity sub-study and will be monitored at participating clinical research sites.

The systems to monitor pregnant and breastfeeding women were successfully in Sisonke and will be expanded in this study, through a pregnancy registry and follow up of birth outcomes by a dedicated safety team.

6. OBJECTIVES AND ENDPOINTS

The **Overall Aim** is to evaluate the **effectiveness, safety** and **immunogenicity** of a heterologous mRNA-1273 boost after either a single dose or two doses of Ad26.COV2.S COVID-19 vaccine among Sisonke participants.

Objectives	Endpoints
**Primary**	
1. To compare the effectiveness of ≥1 dose of Ad26.COV2.S vaccine with a heterologous mRNA-1273 boost on **severe COVID-19** in Sisonke participants with Sisonke participants not boosted with mRNA-1273.	Rates of COVID-19 hospitalizations and deaths among mRNA-1273 boosted Sisonke participants compared to mRNA-1273 unboosted Sisonke participants
2. To compare the effectiveness of ≥1 dose of Ad26.COV2.S vaccine with a heterologous mRNA-1273 boost on **any COVID-19 cases**, with Sisonke participants not boosted with mRNA-1273.	Rates of COVID-19 positive diagnoses (PCR or antigen test positive) among mRNA boosted Sisonke participants versus mRNA unboosted vaccinated populations
**Secondary**	
To compare the effectiveness of a single dose of Ad26.COV2.S vaccine with against without a heterologous mRNA-1273 boost on any COVID-19 infection, severe COVID, hospitalizations and deaths in Sisonke participants.	Rates of COVID-19 infections, hospitalizations and deaths among participants who received a single Ad26.COV2.S dose with or without a mRNA booster
To compare the effectiveness of two doses of Ad26.COV2.S vaccine with against without a heterologous mRNA-1273 boost on any COVID-19 infection, severe COVID, hospitalizations and deaths in Sisonke participants.	Rates of COVID-19 infections, hospitalizations and deaths among participants who received two doses of Ad26.COV2.S with or without a mRNA booster
To compare the effectiveness of a single dose versus two doses of Ad26.COV2.S vaccine with a heterologous mRNA-1273 boost on any COVID-19 infection, severe COVID, hospitalizations and deaths in Sisonke participants.	Rates of COVID-19 infections, hospitalizations and deaths among mRNA boosted Sisonke who received one versus two previous doses of Ad26.COV2.S
To monitor **safety** among participants, including among pregnant and breastfeeding women, receiving a heterologous mRNA-1273 boost either after a single dose or two doses of Ad26.COV2.S vaccine.	All SAEs and adverse events of special interest and birth outcomes will be collected and reported for 6 months after the mRNA-1273 booster vaccination and for up to 1 year among pregnant women.
In a subset of participants (approximately N=200), measure the humoral and cellular immune responses to heterologous mRNA-1273 boost either after a single dose or two doses of Ad26.COV2.S vaccine.	Neutralization titres early (Day 29) and late (Month 6) post-boost versus baseline using pseudovirus and/or live virus neutralization assays. T cell response magnitudes post-boost versus baseline using intracellular cytokine staining.
To monitor the genetic diversity of breakthrough SARS CoV-2 infections	Genetic diversity of breakthrough infection virus as determined by whole genome sequencing. This will be recovered from national laboratories.

7. STUDY DESIGN

This is an open-label, phase 3 COVID-19 heterologous mRNA vaccine booster study nested in the Phase 3b implementation Sisonke implementation trial population among HCWs aged 18 years or older in South Africa. Embedding this trial into the Sisonke study will allow both

an adjusted cohort design (primary analysis) and a matched cohort design comparing Sisonke participants opting for a mRNA booster with Sisonke participants who received only one or two doses of Ad26.COV2.S. Primary and secondary effectiveness endpoints will be collected by utilizing existing national COVID-19 diagnosis and hospitalization records as well as the South African death registry. This study will be conducted by Sisonke (VAC31518COV3012) clinical research sites in South Africa. Sisonke participants registered on the national Electronic Vaccination Data System (EVDS), who had either a single or two doses of the Ad26.COV2.S vaccine will be eligible for enrolment.

The trial will be advertised, and Sisonke participants will be invited for the mRNA-1273 booster vaccination through recruitment activities by the study site staff. Booster vaccinations will be prepared and overseen by clinical trial site personnel. If a participant is unable to complete the study, but has not withdrawn consent, an early exit visit will be conducted. The end-of-study will be considered as at least 6 months and up to 2 years of follow up for the last participant enrolled in the study, or when the Sisonke study ends. Participants will receive an intramuscular injection of mRNA-1273 at enrolment at the recommended booster dose level of 50mcg. Surveillance for effectiveness may continue for up to 2 years post vaccination until 17 May 2024. Surveillance for safety will be for a minimum of 6 month after mRNA-1273 booster vaccination, and up to 1 year among pregnant women.

7.1 Enrolment plan

The target population are HCWs who received the Ad26.COV2.S vaccine as part of the Sisonke trial (approximately N=500 000). These include almost even groups of HCWs who received one dose or two doses of Ad26.COV2.S since February 2021. Volunteers already registered on the Department of Health Electronic Vaccination Data System (EVDS) will be invited to make an appointment at one of the clinical research sites, will be offered to join the study, and sign an informed consent form before receiving the booster vaccine dose.

7.2 Study population

The aim is to enrol up to approximately 15 000 Sisonke participants into two groups who will receive the mRNA booster. These will include HCWs who received two doses of Ad26.COV2.S so far (Group 1) and those that received only one dose of Ad26.COV2.S (Group 2). All participants will be Sisonke participants, 18 years and older, with or without comorbidities including HIV co-infection. People who have received boosting through other means are excluded. Considering the high burden of COVID-19 among pregnant women and the high morbidity, pregnant and breastfeeding women will be included. This inclusion criterion is also supported by global real-world data on the safety of the mRNA-1273 vaccine among pregnant women.

7.3 Eligibility criteria

<u>Inclusion criteria</u>

- Age 18 and older at time of enrolment
- Sisonke participant
- Received a priming Ad26.CoV2.S vaccination as part of the Sisonke study
- If received a second dose of Ad26.CoV2.S vaccine as part of the Sisonke 2 study, this was administered at least 3 months ago
- Participants who are pregnant or report breastfeeding at the time of enrolment may be included.
- Willingness and ability to comply with vaccination plan and other study procedures.
- Capable and willing to provide informed consent

<u>Exclusion criteria</u>

- Participants who have received any COVID-19 vaccines other than one or two doses of Ad26.CoV2.S through other means (for example, another mRNA booster dose).

- Current participation in any other research studies (other than Sisonke) that would interfere with the objectives of this study. The determination of whether participation in another study would be exclusionary for a given participant will be made by the PI/designee.
- Occurrence of a known COVID-19 outcome within 14 days of enrollment
- Participants with a history of heparin-induced thrombocytopenia, or thrombosis and thrombocytopenia syndrome (TTS).
- History of severe adverse reaction associated with a vaccine and/or severe allergic reaction (e.g., anaphylaxis) to any component of mRNA-1273.
- Pregnancy which began prior to or within 30 days after the receipt of dose 2 of Ad26.COV2 and ongoing at the time of enrolment in this trial.
- Acute infection (e.g. febrile illness) unless resolved before enrolment.
- Any significant acute or chronic medical condition, situation or circumstance that in the opinion of the PI/designee makes the participant unsuitable for participation in the study, or jeopardises the safety or rights of the participant

The following participants should only be enrolled after discussion with the PSRT:

- Participants who are thought to have suffered a neurological or cardiac AE considered related to the Ad26.CoV2.S vaccine.
- Participants reporting a non-infective SAE within the first 28 days following the first or second dose of Ad26.CoV2.S vaccine in the Sisonke 3B trial
- History of myocarditis or pericarditis especially in young male
- Chronic history of severe clotting disorders
- Participants who suffered a thromboembolic AE following the Janssen Covid-19 vaccine.

In the event of a clinical contraindication to receiving a heterologous mRNA-1273 booster, the study PSRT, comprising of medical experts in haematology, allergology and neurology supported by safety physicians and experts in vaccine research, will evaluate the medical history of the participant. Based on the medical history and interaction with the participant and/or their medical practitioner, a recommendation will be made with the site PI and the volunteer on how to proceed.

Possible contra-indications include:

- Serious allergic reactions after receiving a previous COVID-19 vaccine
- Venous/arterial thrombosis
- Pulmonary embolus
- Thrombosis and Thrombocytopenia Syndrome (TTS), also known as Vaccine Induced Thrombosis and Thrombocytopenia Syndrome (VITT)
- Immune thrombocytopenia (ITP)
- Guillian Barre Syndrome
- History of capillary leak syndrome
- Any other SAE attributed to previous COVID-19 vaccine

8. CLINICAL PROCEDURES

The study will be conducted at clinical research sites with research staff who worked on the Ensemble trial (Clinical Protocol VAC31518COV3001) and participated in the Sisonke study Ad26.Cov2.S vaccine rollout.

8.1 Recruitment

Recruitment will take place through clinical research site community engagement activities and by advertising the study through existing national communication channels as well as utilizing the National Department of Health's EVDS. Sisonke participants who registered on the EVDS and already had one or two doses of the Ad26.CoV2.S vaccine will be given a date and time for assessment for a booster vaccination through one of the participating research sites. Detailed study information sharing, consent process and eligibility assessment will be conducted at the research sites.

8.2 Screening and enrolment visit into the main study

After providing written consent, participants will be enrolled at designated research sites. Once consented, participants will undergo the eligibility assessment. This will include a clinical assessment. Female participants will have a pregnancy test done to ensure appropriate follow-up can be arranged. Once eligibility is confirmed participants will have limited laboratory procedures done (a nasal swab and a plasma sample for SARS-CoV-2 surveillance) before receiving the vaccine booster. Post-vaccination, participants will be observed for a minimum of 15 minutes.

After the enrolment visit pregnant participants in the main study will be primarily followed up regularly but at least 3-monthly through the Sisonke safety system, which is explained in Section 10. Pregnant and breastfeeding women who are eligible for enrolment will also be followed up by the central safety team, through a pregnancy and breastfeeding registry that both the research sites and safety team have access to. In addition, pregnant and breastfeeding women who are enrolled at clinical research sites that take part in the sub-study, will review these participants as part of the sub-study follow up visits.

The screening and enrolment visits can be conducted as a single visit or two separate visits. (Table 4).

Table 4: Screening and enrolment visit procedures in the main study

**Heterologous mRNA-1273 Boost Study (Main cohort nested in Sisonke)**		
**Visit Number**	**1**	**2**
**Study Day**	**-56 to 1**	**1**
**Procedure**	**Screen**	**Vaccine**
**Study procedures**		
Assessment of Understanding	√	
Informed consent	√	
Medical history	√	
Vaccination history	√	
Physical exam	√	
Obtain demographics	√	
Concomitant medications	√	
Vaccination		√
SAEs, AESIs		√
**Specimen Collections**		
Pregnancy test#	√	
Blood plasma for SARS CoV-2 serology (4 mls EDTA)		√
Nasal swab for SARS CoV-2 PCR		√

Pregnant women will be added to the pregnancy registry of the study and will have an obstetric and gynaecological history recorded. Follow up will be conducted through the sub-study and through the central Sisonke safety desk.

8.3 Screening and enrolment visit into the safety and immunogenicity sub-study

A cohort of approximately 200 participants will be enrolled into the safety and immunogenicity sub-study. After providing written consent, participants will be enrolled at designated research sites. Participants will have a clinical assessment done including a pregnancy test to assess eligibility. At clinical research sites that take part in the safety sub-study, all pregnant women that are enrolled will automatically enrol into the sub-study. Once eligibility has been confirmed baseline immunogenicity bloods will be drawn before the booster vaccine will be administered. Post-vaccination, participants will be observed for a minimum of 15 minutes. The screening and enrolment visits can be conducted as a single visit or two separate visits. (Table 5).

Table 5: Schedule of evaluation for participants in the safety and immunogenicity sub-study (approximately N=200)

**Safety and Immunogenicity Sub-study**				
**Visit Number**	**1**	**2**	**3**	**4**
**Study Week**		0	4	24
**Study Day**	-56 to 1	1	29	169
**Procedure**	**Screen**	**Vaccine**		
**Study procedures**				
Assessment of Understanding	√			
Informed consent	√			
Medical history	√			
Gynaecological and obstetric history*	(√)			
Vaccination history	√			
Physical exam	√			
Obtain demographics	√			
Concomitant medications	√			
Vaccination		√		
SAEs, AESIs assessment		√	√	√
Early reactogenicity assessment		√		
AEs for 28 days post vaccination		√	√	
**Specimen Collections**				
Pregnancy test #	√			
Blood plasma (approx. 8 mls)		√	√	√
Blood PBMC (approx. 42 mls)+		√	√	√
Nasal swab for COVID PCR		√	√	√
Breastmilk^ for immune responses		(√)	(√)	(√)

#female participants only, *pregnant and breastfeeding participants only, ^optional procedure for breastfeeding women, +no PBMC samples will be collected from pregnant women

8.4 Follow up visits in the safety and immunogenicity sub-study

Participants in the safety sub-study will be reviewed after 4 weeks (peak response) and 24 weeks (durability). At the 4-week visit, participants, including pregnant and breastfeeding women, will be reviewed for reactogenicity events and AEs. Any grade 3 or 4 events will be recorded into the database. While AEs will only be recorded for 28 days post-vaccination, AESIs and SAEs will be recorded throughout the study. These will be reviewed weekly by the PSRT.

8.5 Laboratory tests

Plasma and PBMC samples will be collected from the sub-study participants at enrolment, week 4 and week 24 to assess immune responses to the booster vaccine. In addition, nasal swabs will be collected for SARS CoV-2 PCR testing at all visits. Pregnant women will be excluded from the PBMC collection.

8.6 Administration of study product

The single-dose booster vaccine (mRNA-1273) will be prepared by research pharmacists trained on the study protocol. Clinical research staff will then administer the vaccine either in the left or right deltoid as an IM injection only to participants who have met the eligibility criteria. Once vaccinated, participants will be observed for a minimum of 15 minutes for any immediate reactogenicity events or allergic reactions. Any AEs experienced on the day of vaccination will be managed and recorded by the clinical research site teams. Once the observation period is over, and participants are well, they will be issued with a vaccination card, site contact details and contact details for the Sisonke safety desk in case they experience any AEs going forward.

8.7 Surveillance for hospitalization and breakthrough infections

All COVID-19 hospitalisations nationally will be linked with the vaccination register fortnightly to identify whether vaccinated participants have had breakthrough infections. Breakthrough infections will be investigated for severity of infection and outcome. Viral sequencing may also be done by saving the original nasal swab specimen and shipping the swab to the national laboratories who conduct these tests. Medical records and routine laboratory results will be reviewed for participants with AESIs and SAEs.

Unscheduled follow-up visits or contact, e.g. in hospitalised participants may be triggered when a participant has any symptoms of COVID-19. This includes any of the following:
New onset or worsening of any 1 of the symptoms, which lasts for at least 24 hours, not otherwise explained:

- Headache
- Malaise (appetite loss, generally unwell, fatigue, physical weakness)
- Myalgia (muscle pain)
- Chest congestion
- Cough
- Runny nose
- Shortness of breath or difficulty breathing (resting or on exertion)
- Sore throat
- Wheezing
- Eye irritation or discharge
- Chills
- Fever (≥38.0°C or ≥100.4°F)
- Pulse oximetry value ≤95%, which is a decrease from baseline
- Heart rate ≥90 beats/minute at rest, which is an increase from baseline
- Gastrointestinal symptoms (diarrhoea, vomiting, nausea, abdominal pain)
- Neurologic symptoms (numbness, difficulty forming or understanding speech)
- Red or bruised looking toes
- Skin rash
- Taste loss or new/changing sense of smell
- Symptoms of blood clots: pain/cramping, swelling or redness in your legs/calves
- Confusion
- Bluish lips or face
- Clinical suspicion/judgement by investigator of symptoms suggestive for COVID-19

- During unscheduled follow-up visits, a swab will be taken to detect SARS-CoV-2 as per routine protocol from the National Institute of Communicable Diseases/National Health Laboratory Services (see schedule of events and specimen collection).

If they are unable to return to the site with any symptoms or confirmed COVID-19 diagnosis they will be encouraged to see their own health care practitioners for care, but are also requested to let the relevant research site know. They may also be contacted by the Sisonke safety team via telephone or SMS to encourage feedback on side effects, COVID-19 infection, hospitalisation or related issues.

8.8 Surveillance for hospitalization and breakthrough infections

Outcomes will be defined as per FDA guidelines (accessible at https://www.fda.gov/media/137926/download):

Symptomatic COVID-19

- Positive testing by standard RT-PCR assay or Antigen or equivalent testing with symptoms.
- No clinical signs indicative of severe or critical severity

Severe COVID-19

- Positive testing by standard RT-PCR assay or an equivalent test including antigen test and possibly viral load.
- Symptoms suggestive of severe systemic illness with COVID-19, which could include any symptom of moderate illness or shortness of breath at rest, or respiratory distress
- Clinical signs indicative of severe systemic illness with COVID-19, such as respiratory rate ≥ 30 per minute, heart rate ≥ 125 per minute, SpO_2 ≤ 93% on room air at sea level or PaO2/FiO2 < 300
- No criteria for Critical Severity

Critical COVID-19

- Positive testing by standard RT-PCR assay or equivalent test including antigen test and possibly viral load.
- Evidence of critical illness, defined by at least one of the following:
    - Respiratory failure defined based on resource utilization requiring at least one of the following:
        - Endotracheal intubation and mechanical ventilation, oxygen delivered by high flow nasal cannula (heated, humidified, oxygen delivered via reinforced nasal cannula at flow rates > 20 L/min with fraction of delivered oxygen ≥ 0.5), non-invasive positive pressure ventilation, ECMO, or clinical diagnosis of respiratory failure (i.e., clinical need for one of the preceding therapies, but preceding therapies not able to be administered in setting of resource limitation)
    - Shock (defined by systolic blood pressure < 90 mm Hg, or diastolic blood pressure < 60 mm Hg or requiring vasopressors)
    - Multi-organ dysfunction/failure

9. STUDY PRODUCT

The mRNA-1273 COVID-19 vaccine was developed by Moderna, Inc. It is a lipid nanoparticle (LNP) dispersion of a messenger ribonucleic acid (mRNA) encoding the prefusion stabilized

S protein of SARS-CoV-2 formulated in LNPs composed of 4 lipids (1 proprietary and 3 commercially available).

This vaccine has been issued an Emergency Use Authorization (EUA) by the US Food and Drug Administration (FDA) and was granted a conditional marketing authorization (CMA) by the European Commission for active immunization to prevent COVID-19 caused by SARS-CoV-2 virus in individuals 18 years and older. On 31 January 2022, the FDA granted approval for of the Moderna mRNA-1273 vaccine (marketed as Spikevax (COVID-19 Vaccine, mRNA).The vaccine has also been granted Emergency Use Listing (EUL) by the World Health Organization and will be administered after authorization by in-country National Regulatory Authorities for active immunization to prevent COVID-19.

The COVID-19 mRNA-1273 booster vaccine is a suspension for intramuscular injection (IM) administered as a single dose (50 mcg in 0.25 mL).

9.1 Preparation, Handling and Storage

All study vaccine must be appropriately stored in a secured research site pharmacy location and at controlled temperatures as indicated on the clinical labels. If study vaccine is exposed to temperatures outside the specified temperature range, all relevant data will be sent to the Sponsor to determine if the affected supplies can be used or will be replaced. The affected study vaccine must be quarantined and not used until further instruction from the Sponsor is received.

9.2 Study product accountability

The vaccine product will be handled in accordance with the SAHPRA specifications for an investigational product. Potentially hazardous materials containing hazardous liquids, such as needles and syringes should be disposed of immediately in a safe manner and therefore will not be retained for vaccine accountability purposes. Returned study vaccine must not be dispensed again, even to the same participant. Further guidance and information for the final disposition of unused study vaccine are as provided by the Sponsor.

10. CLINICAL SAFETY

Phase 1/2a and Phase 3 studies have shown that the mRNA-1273 vaccine is generally safe to be administered as a homologous and heterologous booster dose in populations in the United States, Canada and Europe. This phase 3 study will gather data on adverse events (AEs), AE of Special Interests (AESIs) and Serious AEs (SAEs), pregnancy and neonatal outcomes and AEs through clinic-based assessment and through the safety surveillance systems that have been put in place during the Sisonke study implementation across South Africa.

The aim is to recruit into the safety and immunogenicity sub-cohort, in parallel to enrolment into the main study population cohort to collect additional safety data at the site visits as well as through the Sisonke safety surveillance system. The safety cohort will be followed with clinic visits up to six months after the booster vaccination. AEs will be reported up to Day 29, and SAEs and AESIs will be reported throughout the study. Pregnancy and neonatal outcomes and AEs will also be assessed and reported as part of the sub-study. In the main study cohort, safety monitoring will take place at the research sites on the day of vaccination after which participants will be monitored for safety through the existing Sisonke surveillance system. Safety monitoring will continue through the surveillance system until the end of the study (approximately 1 year after the last mRNA booster vaccination).

10.1 AE reporting

Sites are expected to notify the Sisonke safety desk staff of any serious safety concerns requiring their attention within 3 business days and site investigators are required to submit AE information in accordance with IRB/EC regulatory requirements.

An AE is any untoward medical occurrence in a clinical investigation participant administered a study product/procedure(s) and which does not necessarily have a causal relationship with this treatment. An AE can therefore be any unfavorable and unintended sign (including an abnormal laboratory finding), symptom, or disease temporally associated with the use of an investigational study product/procedure(s), whether or not related to the investigational study product/procedure(s). All AEs are graded according to the Division of AIDS (DAIDS) Table for Grading the Severity of Adult and Pediatric Adverse Events, Corrected Version 2.1, July 2017, available on the RSC website at https://rsc.niaid.nih.gov/clinical-research-sites/daids-adverse-event-grading-tables, except:

Injection Site Erythema or Redness and Injection Site Induration or Swelling activities such that:

- Grade 1 is: 2.5 to < 5 cm in diameter;
- Grade 2 is: ≥ 5 to < 10 cm in diameter;
- Grade 3 is: ≥ 10 cm in diameter OR Ulceration OR Secondary infection OR Phlebitis OR Sterile abscess OR Drainage;
- Grade 4 is: Potentially life-threatening consequences (eg, abscess, exfoliative dermatitis, necrosis involving dermis or deeper tissue);

Clinic staff should evaluate AEs to determine (1) if the AE meets the criteria for prompt PSRT AE review, (2) the AE meets the requirements for expedited reporting, and (3) if the AE is a potential immune-mediated disease that may be listed as AESI.
For the safety subset of participants, the following data will be collected and reported:

- Unsolicited AEs for 28 days following each vaccination
- Solicited AEs following each vaccination

For all participants, the following AEs will be collected and reported throughout the entire study:

- SAEs/EAEs
- AESIs

The AEs collected in this study are reported to the safety team on the appropriate CRF.

10.2 Serious adverse event

The term “Serious Adverse Event” (SAE) is defined as follows: An adverse event or suspected adverse reaction is considered serious if, in the view of either the investigator or the Sponsor, it results in any of the following outcomes:

- death,
- a life-threatening adverse event,
- inpatient hospitalization or prolongation of existing hospitalization,
- a persistent or significant incapacity or substantial disruption of the ability to conduct normal life functions,
- congenital anomaly/birth defect.
- Is Medically Important*

* Important medical events that may not result in death, be life-threatening, or require hospitalization may be considered serious when, based upon appropriate medical judgment,

they may jeopardize the patient or subject and may require medical or surgical intervention to prevent one of the outcomes listed in this definition.

“Life-threatening” refers to an adverse event that at occurrence represents an immediate risk of death to the subject. Similarly, a hospital admission for an elective procedure is not considered an SAE.

10.3 Adverse Event of Special Interest (AESI)

An AESI is an AE (serious or nonserious) of scientific and medical concern specific to the product or programme. All AESIs will be collected through the entire study period and will be reported as SAEs per institutional guidelines. A list of AESI (including the link where updates may be found) is included in Appendix 1: COVID-19 Updated AESI List – Safety Platform for Emergency vaCcines Project (SPEAC). These AESIs are to be prospectively collected throughout the duration of the study.

10.3.1 Anaphylaxis

All suspected cases of anaphylaxis should be recorded as AESIs and reported as an SAE, based on the criteria for a medically important event, unless the event meets other serious criteria. For reporting purposes, a participant who displays signs or symptoms consistent with anaphylaxis (as below) should be reported as a potential case of anaphylaxis. This is provided as general guidance for investigators and is based on the Brighton Collaboration case definition.

Anaphylaxis is an acute hypersensitive reaction with multi-organ system involvement that can present as, or rapidly progress to, a severe life-threatening reaction. It may occur following exposure to allergens from a variety of sources.

Anaphylaxis is a clinical syndrome characterized by the following:

- Sudden onset AND
- Rapid progression of signs and symptoms AND
- Involves 2 or more organ systems, as follows:
  - **Skin/mucosal:** urticaria (hives), generalized erythema, angioedema, generalized pruritus with skin rash, generalized prickle sensation, red and itchy eyes
  - **Cardiovascular:** measured hypotension, clinical diagnosis of uncompensated shock, loss of consciousness or decreased level of consciousness, evidence of reduced peripheral circulation
  - **Respiratory:** bilateral wheeze (bronchospasm), difficulty breathing, stridor, upper airway swelling (lip, tongue, throat, uvula, or larynx), respiratory distress, persistent dry cough, hoarse voice, sensation of throat closure, sneezing, rhinorrhea
  - **Gastrointestinal:** diarrhea, abdominal pain, nausea, vomiting

10.3.2 Myocarditis/Pericarditis

A case of suspected, probable, or confirmed myocarditis, pericarditis, or myopericarditis should be reported as an AESI, even if it does not meet criteria per the CDC Working Case Definitions. The event should also be reported as an SAE if it meets seriousness criteria.

The CDC Working Case Definitions are provided in Table 6. These definitions are intended to serve as a guide to help reporting of suspected cases of myocarditis, pericarditis, or myopericarditis, but the diagnosis of suspected cases is left to the investigator's clinical judgment.

Table 6: Case Definitions of Probable and Confirmed Myocarditis, Pericarditis, and Myopericarditis (24)

Condition	Definition	
	**Probable Case**	**Confirmed Case**
**Acute myocarditis**	Presence of ≥ 1 new or worsening of the following clinical symptoms:<br>• Chest pain, pressure, or discomfort<br>• Dyspnea, shortness of breath, or pain with breathing<br>• Palpitations<br>• Syncope<br>**OR** infants and children aged < 12 years might instead have ≥ 2 of the following symptoms:<br>• Irritability<br>• Vomiting<br>• Poor feeding<br>• Tachycardia<br>• Lethargy<br>**AND** ≥ 1 new finding of:<br>• Troponin level above upper limit of normal (any type of troponin)<br>• Abnormal electrocardiogram (ECG or EKG) or rhythm monitoring findings consistent with myocarditis<br>• Abnormal cardiac function or wall motion abnormalities on echocardiogram<br>• cMRI findings consistent with myocarditis<br>**AND**<br>• No other identifiable cause of the symptoms and findings	Presence of ≥ 1 new or worsening of the following clinical symptoms:<br>• Chest pain, pressure, or discomfort<br>• Dyspnea, shortness of breath, or pain with breathing<br>• Palpitations<br>• Syncope<br>**OR** infants and children aged < 12 years might instead have ≥ 2 of the following symptoms:<br>• Irritability<br>• Vomiting<br>• Poor feeding<br>• Tachycardia<br>• Lethargy<br>**AND** ≥ 1 new finding of:<br>• Histopathologic confirmation of myocarditis<br>• cMRI findings consistent with myocarditis in the presence of troponin level above upper limit of normal (any type of troponin)<br>**AND**<br>• No other identifiable cause of the symptoms and findings
**Acute pericarditis****	Presence of ≥ 2 new or worsening of the following clinical features:<br>• Acute chest pain<br>• Pericardial rub on examination<br>• New ST-elevation or PR-depression on EKG<br>• New or worsening pericardial effusion on echocardiogram or MRI	
**Myopericarditis**	This term may be used for patients who meet criteria for both myocarditis and pericarditis.	

10.4 Expedited reporting of adverse events

Clinical research site staff must report all related SAEs and AESIs within 24 hours of their knowledge of the event, and any updated information about a previously reported event to the clinical database and the sponsor, so that applicable safety data can be reviewed by the safety team.

10.5 Safety surveillance in the main study

A safety surveillance system has been set up and successfully implemented and tested during the Sisonke study (Figure 1).

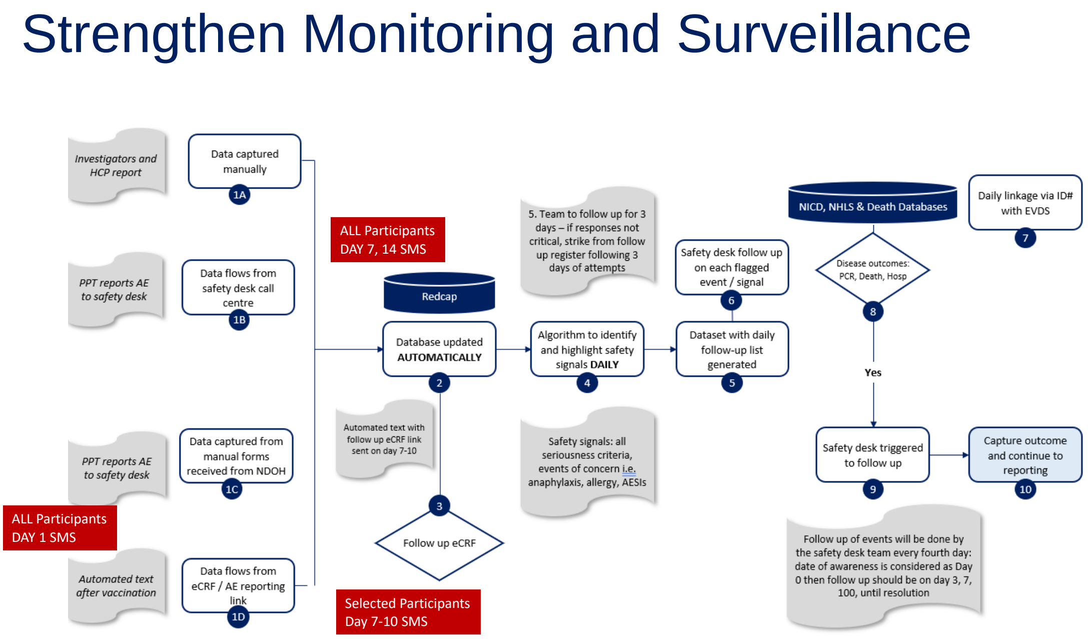

Figure 1: Overview of the pharmacovigilance and AE reporting process in the Sisonke study.

In the main study, up to approximately 15 000 participants, will receive the mRNA-1273 vaccine at the research site and will be monitored for a minimum of 15 minutes. Participants will then receive text messages on day 7 and 28 after vaccination with messaging on common signs and symptoms of reactogenicity. This message will include a link to an AE reporting form, which will automatically be transmitted to the study safety desk database and will be screened by safety staff. In addition, study participants will have access to the toll-free phone number for Sisonke Safety desk to report any symptoms. The Sisonke safety team will receive a daily report for them to follow up, initiate causality assessment and update the study database. Contact details for the safety desk are also available on the vaccination cards and are included in the SMS sent. Vaccinees are reminded of this facility after their vaccination and are again invited to call in to the safety desk if they have a reportable event/ side effect. The safety team will then prepare reports for the sponsor, manufacturer and SAHPRA per reporting timeframe agreement.

Linkage with National Disease Databases: linkage of Sisonke study data with the NHLS Corporate Datawarehouse/ COVID-19 PCR line listing, DATCOV (COVID-19 related

hospitalizations) list and MRC Burden of Disease Unit/Home Affairs death registration list. This aims to identify vaccinees with the study disease outcomes, namely SARS CoV-2 PCR positivity, COVID-19 related hospitalisation and COVID-related deaths.

Adverse events in the database are reviewed daily and mined for potential safety signals: hospitalisations, potentially life-threatening events, and events of medical concern (including pre-defined adverse events of special interest). These are flagged and depending on the nature of the event – pharmacovigilance nurses or the safety physicians follow a prescribed schedule to follow up these participants.

10.6 Summary of post vaccination safety monitoring and reporting in the main study

1. All participants will be assessed and monitored at the research site on the day of vaccination.
2. Participants are then encouraged to report their AEs via the AE electronic reporting system or the toll free phone number to the Sisonke safety desk and will be prompted by the SMSs. The safety desk call number will be provided to the participants.
3. Participants are asked to report to the safety desk should any event occur up to the end of the study follow up period, as Sisonke study follow up continues.
4. PSRT may be convened at any time urgently to discuss SAEs or AESIs including anaphylaxis events, if they occur.
5. Weekly PSRT meetings will be scheduled
6. COVID-19 related hospitalisations are tracked through the DATCOV hospital surveillance system and linked using the unique identifier.
7. All deaths will be tracked through linkages with the MRC Burden of Disease Unit - Home Affairs Death Registration list using the unique identifier for periodic comparison of all-cause mortality to background population mortality rates.

10.7 Safety of pregnant women

Pregnancy testing will be conducted at the screening/enrolment visit for all female participants of reproductive age in the sub-study and main cohort. In addition, there may be a small number of participants who are already pregnant at entry to the study. If a pregnancy is detected, and other eligibility criteria are met, the participant will have an obstetric and gynaecological history taken and will be recorded as a pregnancy in the study database. These data will be automatically linked to the study safety team who will be monitoring the pregnancy registry. Safety follow up for adverse pregnancy or birth outcomes will then be conducted by a dedicated team at the Sisonke safety desk with assistance, where necessary, by site-PIs. This will involve communication with participants and collecting and summarizing medical records, if necessary. Contacts with pregnant participants will take place at least 3-monthly. Pregnancy reports will be prepared periodically and will be reported together with the general safety report for the study to the Sponsor, Moderna and SAHPRA. Pregnant participants will be followed up for maternal and birth outcomes.

Furthermore, at study sites that contribute to the safety and immunogenicity sub-study, women who are pregnant or breastfeeding at enrolment will enrol into the sub-study and will continue with sub-study visits as per schedule of evaluation. At follow-up visits, participants will be assessed for any AEs related to pregnancy and birth outcomes. Birth outcomes will be collected beyond the 6-month sub-study exit visit, by study sites staff and the safety team.

10.8 Safety Oversight

10.8.1 Safety Monitoring Committee

The safety monitoring committee is a group of independent experts with cumulative experience in ethics, infectious diseases, COVID-19, vaccine clinical trials and epidemiology/biostatistics who will provide independent oversight of the study. They will convene frequently in open forum.

Current Members of the Sisonke Safety Monitoring Committee will be invited to join the SMC for this trial nested in Sisonke:

- Chris Beyrer: USA (Epidemiology and vaccinology)
- Francesca Conradie: RSA (Infectious disease)
- Sipho Dlamini: RSA (Infectious disease and vaccinology)
- Jeremy Nel: RSA (Infectious disease)
- Yunus Moosa: RSA (infectious disease)

10.8.2 Protocol Safety Review Team

Safety monitoring and oversight will be provided by the Sisonke protocol safety review team (PSRT). The PSRT is composed of the following members:

- PI and co-PIs
- Safety physicians/medical monitors and medical officers from Sponsor and collaborator
- Representatives from clinical specialities, including Immunology, Haematology, Neurology, Cardiologist and Obstetrician and Gynaecologist

The members of the Sisonke PSRT are responsible for decisions related to participant safety. Other protocol team members, site investigators, external experts may also be included in PSRT meetings. The PSRT will review cumulative clinical safety data on a weekly basis. The PSRT will include the following members:

Sisonke Safety Desk	Co- opted expert members	Investigators
• Sisonke Safety Physicians<br>• Sisonke Safety Medical Officers	• Clinical Immunology and Allergies<br>• Clinical Haematology<br>• Neurology<br>• Cardiology<br>• Obstetrician and Gynaecologist	• Sisonke Protocol Team Leadership<br>• Moderna Safety Experts

10.8.3 Clinical Safety staff

Clinical safety staff at the HCRISA safety desk comprise safety physicians/medical officers and a team of pharmacovigilance officers (registered nurses and/or clinical associates). The roles and responsibilities of the HCRISA safety desk in relation to safety monitoring include:

- Monitoring and follow-up of safety clinical concerns via a centralized toll-free telephone service
- Monitoring of data for safety and adverse events
- Liaison between clinical research sites and the protocol PSRT/ Manufacturer on clinical safety issues and provide support to the PSRT
- Notifying clinical research sites and other groups of important safety concerns
- Periodic linkage with EVDS, NHLS CDW database and DATCOV via unique identifiers to track safety events
- Track pregnant and breastfeeding participants and pregnancy outcomes

- Management of safety reporting to the PSRT, ethics committees, PIs, collaborators and SAHPRA
- Coordination of collaborative activities with site staff for follow up and retrieval of medical records

10.9 Safety reporting and review

10.9.1 Safety reporting

The safety physician and the PSRT will ensure all safety events that require notification are reported to SAHPRA and the Manufacturer according to the applicable regulatory requirements.
The data elements collected during safety monitoring will include:

- AEs until 28 days after administration of the booster dose of the study product (sub-study)
- AESIs and SAEs until the end of the study.
- Disease-related events and outcomes per self-report or as identified via periodic linkage to the following databases: These include COVID-19 infection, COVID-19 related disease, hospitalisations and deaths.
  - Routine and diagnostic specimen data from the national laboratory management information system are archived in the Corporate Data Warehouse (CDW) of the NHLS and COVID-19 related hospitalisations are monitored via the Daily Hospital Surveillance system (DATCOV). The databases will be scanned for potential new COVID-19 infections or severe adverse events/hospitalisations and evidence of COVID-19 like disease as per participant permission.
  - Causes of deaths and mortality rates will be ascertained via family or health facility/hospital report, active tracing and the SAMRC Burden of Disease linkage with the South African National Vital Registration Infrastructure Initiative.

10.9.2 Review of cumulative safety data

The PSRT will review periodic cumulative safety data. SAHPRA will be provided with regular periodic reports per regulatory requirements including safety and futility reports and regular progress reports. Routine safety review occurs at the start of enrollment and then throughout the study. Reviews proceed from a standardized set of protocol-specific safety data reports. These reports are produced by the safety team.

11. STATISTICAL CONSIDERATIONS

11.1 Study design

This is an open-label phase 3 vaccine study. The effect of the mRNA-1273 booster vaccine will be analysed using two statistical approaches: 1) the cohort approach adjusted for potential confounders (Figure 2) and 2) the matched cohort approach (Figure 3). Within the cohort analysis approach, two cohorts will be modeled separately: 1a) The Sisonke cohort of approximately 477 000 individuals and 1b) the sub-cohort of approximately 15 000 mRNA-1273 boost recipients. The primary analysis approach will be the adjusted cohort approach which includes all Sisonke participants (Approach 1a), while the sub-cohort approach (Approach 1b) will be a sub-analysis detailed in the statistical analysis plan. The two primary endpoints of severe COVID-19 (hospitalizations and deaths) and any COVID-19 infection will be analysed.

11.2 Statistical analyses

Analyses for primary endpoint(s) and some of the secondary endpoints will be performed using SAS version 9.4 (Statistical Analysis Software, North Carolina, USA) and R statistical software. All HCWs will be included in the analyses aimed at measuring vaccine uptake.
All deviations to be made to the statistical considerations in this protocol will be documented in the detailed statistical analyses plan (SAP) together with a detailed analysis plan for secondary objectives.

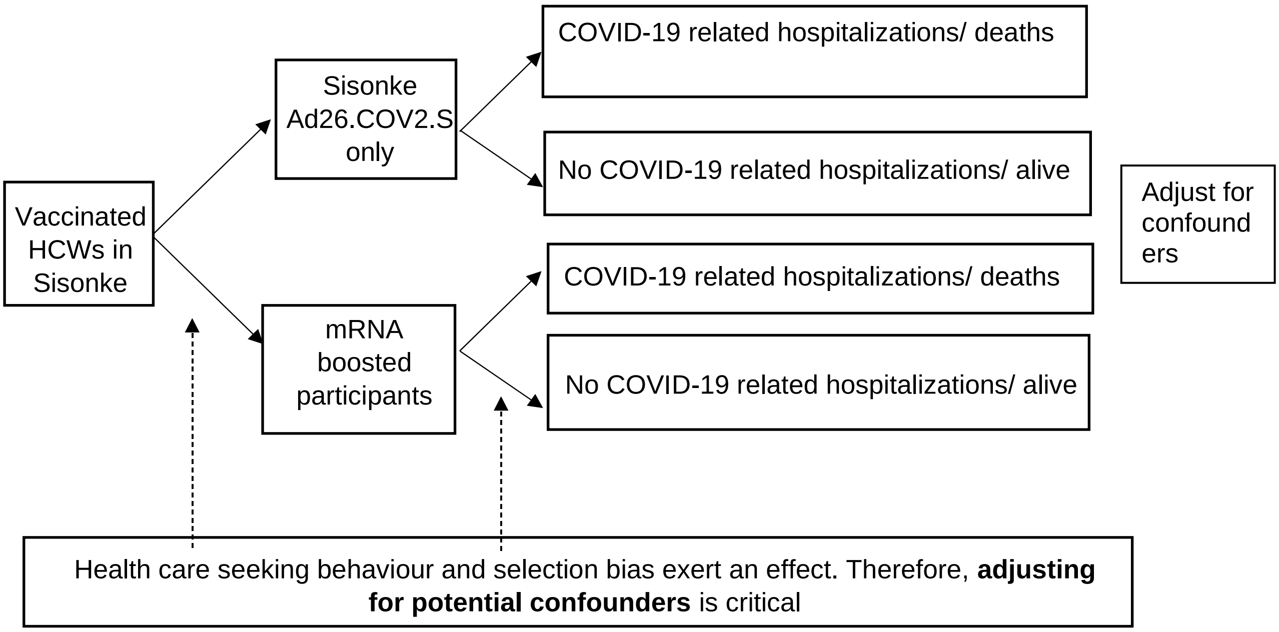

Figure 2: Flow diagram showing the adjusted cohort study design.

<u>Participant demographics and baseline clinical data</u>
Demographic and clinical data of all participants enrolled in the study will be summarized using descriptive statistics.

<u>Analyses of the primary endpoint</u>
For the primary analysis approach (i.e the adjusted cohort approach on the Sisonke cohort), a Cox regression model will be used with booster (mRNA-1273) status as a time-varying covariate adjusting for the following variables 'age, gender, month of first (and second) Ad26.COV2.S vaccination, geographic location, number of comorbidities and evidence of prior COVID-19 infection'. Further details regarding the definitions of the aforementioned variables will be detailed in the SAP This time-varying exposure will be characterized by a continuous piecewise linear function of time elapsed since the first dose for the log hazard ratio, with a change point placed at every month. The VE in reducing the risk of severe disease will be estimated as one minus the estimated hazard ratio; corresponding 95% confidence intervals will be constructed.

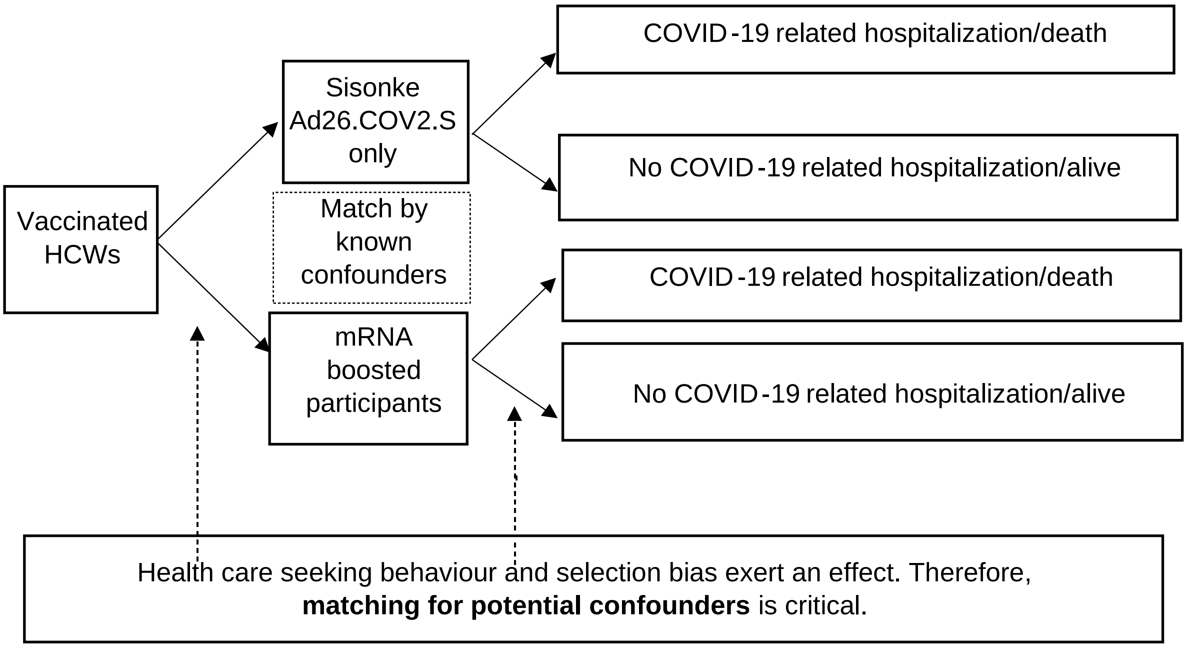

Figure 3: Flow diagram showing the matched cohort study design.

In the secondary analysis approach, non-mRNA boost recipients and mRNA boost recipients will be matched using propensity scores, estimated from logistic regression. The following variables may be included: age, gender, month of first (and second) Ad26.COV2.S vaccination, geographic location, number of comorbidities and evidence of prior COVID-19 infection. The index date for each propensity score matched pair is the calendar date of mRNA booster dose vaccination. A person can contribute both unboosted and boosted person-time, if boosted during the course of follow-up. Data for boosted and unboosted matched pair will be censored when the unboosted individual eventually receives a vaccine booster dose. The VE will be estimated as one minus the estimated hazard ratio; corresponding 95% confidence intervals will be constructed.

Safety data (hospitalizations and/or deaths)
The number and the proportion of hospitalized or died due to COVID-19 will be reported for all Sisonke participants, and, where necessary, these results will be stratified by province, age, gender and co-morbidity status.

11.3 Sample size calculations

Sample size calculations for the primary endpoint are based on the log rank test. In the ongoing Sisonke cohort analyses, an attack rate of 0.3% (i.e. COVID-19 hospitalizations or deaths) was observed among vaccinated individuals.

11.3.1 Adjusted cohort analysis - Primary analysis

Under the adjusted cohort approach, all 477 000 Sisonke participants will be analysed, of whom approximately 15 000 are mRNA-1273 boosted. Under this approach, with the aforementioned group sizes, an attack rate of 0.3%, a VE of 42% or greater against severe disease (associated with observing 1413 events) can be detected with 80% power (Table 7). Under this approach, an attack rate of 5%, a VE of 10.4% or greater against any infection (associated with observing 23 786 events) can be detected with 80% power (Table 8).

Table 7: Sample size scenarios for different number of severe COVID-19 events and booster vaccine effectiveness

Total sample size	Sample size mRNA boost	Sample size non-mRNA boost	Number of events	VE	Attack rate in non-mRNA boost	Power
477234	15000	462234	1410	50%	0.30%	91%
477234	15000	462234	1412	45%	0.30%	85%
**477234**	**15000**	**462234**	**1413**	**42%**	**0.30%**	**80%**
477234	15000	462234	1414	40%	0.30%	76%
477234	15000	462234	1416	35%	0.30%	64%

Table 8: Sample size scenarios for different number of any COVID-19 infection and booster vaccine effectiveness

Total sample size	Sample size mRNA boost	Sample size non-mRNA boost	Number of events	VE	Attack rate in non-mRNA boost	Power
477234	15000	462234	23492	50%	5%	>99%
477234	15000	462234	23567	40%	5%	>99%
477234	15000	462234	23641	30%	5%	>99%
477234	**15000**	462234	23678	25%	5%	>99%
477234	15000	462234	23715	20%	5%	>99%
477234	15000	462234	23752	**15%**	5%	98%
477234	15000	462234	23789	10%	5%	77%

11.3.2 Matched cohort analysis - Secondary analysis

Under the matched cohort approach, Table 9 shows the number of individuals required in each arm, under VE for severe COVID-19 ranging from 35 - 50% and a ratio of individuals of 4:1 for non-mRNA boosted to mRNA boosted, based on a type 1 error rate of 5% and power of 80%. Based on these calculations a sample size of 13894 in the mRNA-1273 boost arm will allow sufficient power to detect at least a VE of 40%.

Table 9: Sample size scenarios for different number of severe COVID-19 events and booster vaccine effectiveness

Total sample size	Sample size mRNA boost	Sample size non-mRNA boost	Ratio (non-mRNA to mRNA)	Number of events	VE	Attack rate in non-mRNA boost
39225	7845	31380	4	71	50%	0.30%
51657	10332	41325	4	100	45%	0.30%
**69467**	**13894**	**55573**	**4**	**142**	**40%**	**0.30%**
96073	19215	76858	4	208	35%	0.30%

For the second primary endpoint of any COVID-19, the sample size calculation assumes an attack rate of 5%. A sample size of 15000 in the mRNA-1273 boost arms confers more than 96% power to detect VE of 15% or greater.

12. DATA MANAGEMENT

12.1 Overview of data management

All data management activities will be undertaken under the applicable regulatory frameworks. This includes the U.S Food and Drug Administration (FDA) regulations, European Medicines Agency (EMA) regulations and the SAHPRA regulations. All studies will also abide by the Research Ethics Committee regulations of participating institutions. The Data management systems at the Centre for the AIDS Programme of South Africa (CAPRISA) and the SAMRC meet FDA requirements as they are CFR Part 11 and POPIA compliant. The Data Management standard processes are aligned with the Good Clinical Data Management Processes (GCDMP).

Data will be collected on electronic questionnaires which will be developed and managed by the study team. All site study staff will be trained on the correct completion of the questionnaires. If data entered on the questionnaires are taken from an external source (e.g., laboratory reports, participant records), the source documents will be maintained in the participant's medical chart or study file at the site and will be available for review. The questionnaire will then be captured into the electronic data management system.

All data will be captured on a Web-based Electronic Data Capture System and will be accessible to the study staff and the statistician via controlled password access. The data management team will have read write-access, with audit logging controlled by passwords and access validation levels. Study staff who have access to the data on the computer systems will be trained on how to access the system.

The electronic data management system will use prescribed API (Application Programming Interface) which can be set with conditional access for all inter operable integrations and data sharing as and when required.

The system will be housed in a secure data centre with restricted access control and all relevant environmental measures to ensure the integrity of the Servers. Hourly and Daily backups are created and stored in various locations in accordance to the CAPRISA Disaster Recovery (DR) plan. Physical access to the Servers and Operating systems are maintained by Information Technology department.

12.2 Data analyses

<u>Surveillance:</u> Automated near real-time deterministic and probabilistic linkage algorithms will be performed to link three routine COVID-19 datasets:

i. Notifiable Medical Conditions Sentinel Surveillance (NMCSS) database of SARS-CoV-2 cases
ii. DATCOV hospital database of reported confirmed COVID-19 hospital admissions
iii. The national SARS-CoV-2 vaccine registry

<u>Breakthrough infections,</u> defined as a positive SARS-CoV-2 PCR or antigen test ≥7 days after completion of vaccination, and hospitalisations will be identified through linking the national vaccine registry with NMCSS and DATCOV databases, respectively. Analysis of breakthrough infections will occur in those Sisonke participants who have been hospitalised or died.

12.3 Data Sharing

The study team scientists will disseminate the trial results as broadly as possible. Data will be shared with Moderna and JnJ prior to release to the public. The study data will be published consistent with normal scientific practices. Research data that document, support, and validate research findings will be made available after the main findings from the final research dataset have been accepted for publication. Such research data will be modified to prevent the disclosure of personal identifiers to remain in compliance with the Protection of Human Subjects. The research team will attend conferences periodically and present trial results to a

multidisciplinary scientific community. The results from this research may also be disseminated through presentations at scientific institutions/ meetings, and/or publication in scientific journals. All publications will be uploaded to a publication repository. After sharing the results with study participants, they will be presented to communities from which participants are recruited, following Good Participatory Practice guidelines. The results will also be shared with global and local policy makers. Summary results of the trial will be made publicly available through the clinical trial registry. Any datasets used for analysis in publications can be requested by investigators via an online request to the organisation. Measures will be taken to protect identifiable information in the datasets.

13. HUMAN SUBJECT PROTECTION AND ETHICAL OBLIGATIONS

13.1 Regulatory and Ethical Approval

The study will be conducted in accordance with all conditions of approval by the relevant regulatory authorities and ethics committee in South Africa.

13.2 Informed consent

Written Informed Consent will be obtained prior to enrolment into the study and prior to vaccination.

13.3 Risks and Benefits

Subject confidentiality

Every effort will be made to protect participant privacy and confidentiality to the extent permitted by law.

Study discontinuation

This study may be discontinued at any time if approval for the study is withdrawn by regulatory authorities and ethics committees.

13.4 Monitoring

Clinical Research Site monitoring is conducted by Hutchinson Centre Research Institute of South Africa (HCRISA) to ensure that the rights and well-being of trial participants are protected, that the reported trial data are accurate, complete, and verifiable, and that the conduct of the trial is in compliance with the currently approved protocol/amendment(s), Declaration of Helsinki, Guidelines for Good Practice in the Conduct of Clinical Trials in Human Participants in South Africa, and with applicable regulatory requirement(s). Refer to Sisonke Phase 3b Open Label Clinical Trial Monitoring Plan for more information about monitoring schedule, reports, virtual and onsite support.

14. REFERENCES

1. Sadoff J, Gray G, Vandebosch A, et al. Final Analysis of Efficacy and Safety of Single-DoseAd26.COV2.S. ENSEMBLE Study Group.N Engl J Med. 2022 Feb 9. doi: 10.1056/NEJMoa2117608.

2. Linda-Gail Bekker, Nigel Garrett, Ameena Goga, et al. Effectiveness of the Ad26.COV2.S vaccine in health care workers in South Africa. The Lancet (in print) Pre-print available at https://papers.ssrn.com/sol3/papers.cfm?abstract_id=3979291
3. Takuva S, Takalani A, Garrett N, et al. Thromboembolic Events in the South African Ad26.COV2.S Vaccine Study. N Engl J Med. 2021 Aug 5;385(6):570-571. doi: 10.1056/NEJMc2107920.
4. Simbarashe Takuva, Azwidhwi Takalani, Ishen Seocharan, et al. Safety of the single-dose Ad26.CoV2.S vaccine among healthcare workers in the phase 3b Sisonke study in South Africa. Available at https://www.medrxiv.org/content/10.1101/2021.12.20.21267967v1
5. Glenda E Gray, Shirley Collie, Nigel Garrett, et al. Vaccine effectiveness against hospital admission in South African health care workers who received a homologous booster of Ad26.COV2 during an Omicron COVID19 wave: Preliminary Results of the Sisonke 2 Study. Available at https://www.medrxiv.org/content/10.1101/2021.12.28.21268436v1
6. Atmar RL, Lyke KE, Deming ME, et al. Homologous and Heterologous Covid-19 Booster Vaccinations. N Engl J Med. 2022 Jan 26:NEJMoa2116414. doi: 10.1056/NEJMoa2116414.
7. U.S. Food and Drug Administration (FDA). Moderna COVID-19 Vaccine EUA Letter of Authorization 2020. Available at https://www.fda.gov/media/144636/download
8. U.S. Food and Drug Administration (FDA). Pfizer-BioNTech COVID-19 Vaccine EUA Letter of Authorization 2020 Available at https://www.fda.gov/media/144412/download.
9. Baden LR, El Sahly HM, Essink B, et al. Efficacy and Safety of the mRNA-1273 SARS-CoV-2 Vaccine. N Engl J Med. 2021;384(5):403-16.
10. Lopez Bernal J, Andrews N, Gower C, et al. Effectiveness of Covid-19 Vaccines against the B.1.617.2 (Delta) Variant. N Engl J Med. 2021;385(7):585-94.
11. Hall VJ, Foulkes S, Saei A, et al. COVID-19 vaccine coverage in health-care workers in England and effectiveness of BNT162b2 mRNA vaccine against infection (SIREN): a prospective, multicentre, cohort study. The Lancet. DOI:https://doi.org/10.1016/S0140-6736(21)00790-X
12. Self WH, Tenforde MW, Rhoads JP, et al. Comparative Effectiveness of Moderna, Pfizer-BioNTech, and Janssen (Johnson & Johnson) Vaccines in Preventing COVID-19 Hospitalizations Among Adults Without Immunocompromising Conditions — United States, March–August 2021. Morbidity and Mortality Weekly Report. 2021.
13. El Sahly HM, Baden LR, Essink B, et al. Efficacy of the mRNA-1273 SARS-CoV-2 Vaccine at Completion of Blinded Phase. N Engl J Med 2021; 385:1774-1785. DOI: 10.1056/NEJMoa2113017
14. Polack FP, Thomas SJ, Kitchin N, et al. Safety and Efficacy of the BNT162b2 mRNA Covid-19 Vaccine. New England Journal of Medicine. 2020;383(27):2603-15.
15. VAERS. Vaccine Adverse Event Reporting System 2021. Available at https://vaers.hhs.gov/.
16. Chapin-Bardales J, Gee J, Myers T. Reactogenicity Following Receipt of mRNA-Based COVID-19 Vaccines. JAMA. 2021;325(21):2201-2202. doi:10.1001/jama.2021.5374

17. CDC. Advisory Committee on Immunization Practices (ACIP) Meeting 2021 [updated June 25, 2021. Available at https://www.cdc.gov/vaccines/acip/meetings/slides-2021-06.html

18. Bozkurt B, Kamat I, Hotez PJ. Myocarditis With COVID-19 mRNA Vaccines. Circulation. 2021;144:471–484

19. Singer ME, Taub IB, Kaelber DC. Risk of Myocarditis from COVID-19 Infection in People Under Age 20: A Population-Based Analysis. Available at https://www.medrxiv.org/content/10.1101/2021.07.23.21260998v1.full.pdf

20. Hause AM BJ, Gee J, et al. Safety Monitoring of an Additional Dose of COVID-19 Vaccine — United States, August 12–September 19, 2021.; 2021.

21. Yap M, Debenham L, Kew T, Chatterjee SR, Clinical manifestations, prevalence, risk factors, outcomes, transmission, diagnosis and treatment of COVID-19 in pregnancy and postpartum: a living systematic review protocol. BMJ Open. 2020 Dec 2;10(12):e041868. doi: 10.1136/bmjopen-2020-041868

22. Tom T. Shimabukuro, M.D., Shin Y. Kim, M.P.H., Tanya R. Myers Preliminary Findings of mRNA Covid-19 Vaccine Safety in Pregnant Persons. N Engl J Med 2021; 384:2273-2282. DOI: 10.1056/NEJMoa2104983

23. Kharbanda EO, Haapala J, DeSilva M, et al. Spontaneous Abortion Following COVID-19 Vaccination During Pregnancy. JAMA. 2021;326(16):1629–1631. doi:10.1001/jama.2021.15494

24. Gargano JW, et al. MMWR Morb Mortal Wkly Rep. 2021.

15. Appendices

Appendix 1: COVID-19 Updated AESI List – Safety Platform for Emergency vACcines Project (SPEAC)

Link to updated list: https://brightoncollaboration.us/wp-content/uploads/2021/01/COVID-19-updated-AESI-list.pdf

Medical Concept	Additional Notes
Anosmia, Ageusia	New onset COVID-associated or idiopathic events without other etiology excluding congenital etiologies or trauma.
Subacute thyroiditis	Including but not limited to events of atrophic thyroiditis, autoimmune thyroiditis, immune-mediated thyroiditis, silent thyroiditis, thyrotoxicosis, and thyroiditis.
Acute pancreatitis	• Including but not limited to events of autoimmune pancreatitis, immune-mediated pancreatitis, ischemic pancreatitis, edematous pancreatitis, pancreatitis, acute pancreatitis, hemorrhagic pancreatitis, necrotizing pancreatitis, viral pancreatitis, and subacute pancreatitis.<br>• Excluding known etiologic causes of pancreatitis (alcohol, gallstones, trauma, recent invasive procedures).
Appendicitis	Include any event of appendicitis.
Rhabdomyolysis	New onset rhabdomyolysis without known etiology such as excessive exercise or trauma.
Acute respiratory distress syndrome (ARDS)	Including but not limited to new events of ARDS and respiratory failure.
Coagulation disorders	Including but not limited to thromboembolic and bleeding disorders, disseminated intravascular coagulation, pulmonary embolism, and deep vein thrombosis.
Acute cardiovascular injury	Including but not limited to myocarditis, pericarditis, microangiopathy, coronary artery disease, arrhythmia, stress cardiomyopathy, heart failure, or acute myocardial infarction.
Acute kidney injury	• Include events with idiopathic or autoimmune etiologies<br>• Exclude events with clear alternate etiology (trauma, infection, tumor, or iatrogenic causes such as medications or radiocontrast agents, etc)<br>• Include all cases that meet the following criteria:<br>o Increase in serum creatinine by ≥ 0.3 mg/dL (≥ 26.5 µmol/L) within 48 hours; OR<br>o Increase in serum creatinine to ≥ 1.5 times baseline, known or presumed to have occurred within prior 7 days; OR<br>o Urine volume ≤ 0.5 mL/kg/hour for 6 hours.

Medical Concept	Additional Notes
Acute liver injury	• Include events with idiopathic or autoimmune etiologies<br>• Exclude events with clear alternate etiology (trauma, infection, tumor, etc)<br>• Include all cases that meet the following criteria:<br>o > 3-fold elevation above the upper normal limit for ALT or AST OR<br>o > 2-fold elevation above the upper normal limit for total serum bilirubin or gamma glutamyl transferase or alkaline phosphatase.
Dermatologic findings	• Chilblain-like lesions;<br>• Single organ cutaneous vasculitis;<br>• Erythema multiforme;<br>• Bullous rashes;<br>• Severe cutaneous adverse reactions including but not limited to: Stevens-Johnson syndrome, Toxic Epidermal Necrolysis, Drug Reaction with Eosinophilia and Systemic Symptoms, and fixed drug eruptions.
Multisystem inflammatory disorders	• Multisystem inflammatory syndrome in adults<br>• Multisystem inflammatory syndrome in children<br>• Kawasaki's disease
Thrombocytopenia	• Platelet counts < 150 $\times 10^9$ per mm^3<br>• Including but not limited to immune thrombocytopenia, platelet production decreased, thrombocytopenia, thrombocytopenic purpura, thrombotic thrombocytopenic purpura, or hemolysis, elevated liver enzymes, low platelet count(HELLP) syndrome.
Acute aseptic arthritis	• New onset aseptic arthritis without clear alternate etiology (eg, gout, osteoarthritis, and trauma).
New onset of or worsening of neurologic disease	• Including but not limited to<br>o Guillain-Barre syndrome<br>o Acute disseminated encephalomyelitis<br>o Peripheral facial nerve palsy (Bell's palsy)<br>o Transverse myelitis<br>o Encephalitis/Encephalomyelitis<br>o Aseptic meningitis<br>o Febrile seizures<br>o Generalized seizures/convulsions<br>o Stroke (Hemorrhagic and non-hemorrhagic)<br>o Narcolepsy
Anaphylaxis	• Anaphylaxis as defined per Section **Error! Reference source not found.**.<br>• Follow reporting procedures per Section **Error! Reference source not found.**

Medical Concept	Additional Notes
Other syndromes	• Fibromyalgia<br>• Postural Orthostatic Tachycardia Syndrome<br>• Chronic Fatigue Syndrome (includes myalgic encephalomyelitis and postviral fatigue syndrome)<br>• Myasthenia gravis
