# Supplementary material for: Safety, effectiveness and immunogenicity of heterologous mRNA-1273 boost after prime with Ad26.COV2.S among healthcare workers in South Africa: The single-arm, open-label, phase 3 SHERPA study
Source: PLOS Glob Public Health. 2024 Dec 5;4(12):e0003260. doi: 10.1371/journal.pgph.0003260 (PMC11620404; doi:10.1371/journal.pgph.0003260)
Supplement: S1 Text — (DOCX) [file pgph.0003260.s018.docx]

**Immunogenicity Sub-study Laboratory Assays**

Plasma and PBMCs were collected from a subset of participants at baseline (BL), four weeks (W4) and six months (W24) after the mRNA-1273 booster vaccination. Plasma was used to assess antibody neutralization, binding and antibody dependent cellular cytotoxicity (ADCC). To identify individuals with prior infection or breakthrough infections (BTI) during the study period, we tested all samples for binding against viral nucleocapsid. Of the participants tested, 12/59 and 5/67 of the PLWH were seronegative at BL. When individuals were stratified by Ad26.COV2.S dose, only one participant was seronegative in the one dose Ad26.COV2.S HIV-negative group. Given these small numbers of seronegative participants per group, antibody immunogenicity analyses were only conducted in individuals who were BL seropositive.

*Lentiviral pseudovirus production and neutralization assay*

SARS-CoV-2 pseudotyped lentiviruses were prepared by co-transfecting the HEK 293T cell line with either the SARS-CoV-2 D614G WT (D614G) or Omicron BA.4 (T19I, L24S, Δ25-27, Δ69-70, G142D, V213G, G339D, S371F, S373P, S375F, T376A, D405N, R408S, K417N, N440K, L452R, S477N, T478K, E484A, F486V, Q498R, N501Y, Y505H, D614G, H655Y, N679K, P681H, N764K, D796Y, Q954H, N969K) spike plasmids in conjunction with a firefly luciferase encoding lentivirus HIV-1 pNL4.luc backbone plasmid and incubated at 37 ^o^C for 72 hours. Culture supernatants were clarified using a 0.45-μM filter and stored at −80 °C. For the neutralization assay, the SARS-CoV-2 pseudotyped virus and serially diluted serum samples were incubated for 1 hour at 37°C, 5% CO_2_. Subsequently, 1x10^4^ HEK 293T cells engineered to over-express ACE-2 (293T/ACE2.MF) (kindly provided by M. Farzan (Scripps Research)) were added and incubated at 37°C, 5% CO_2_ for 72 hours upon which luminescence was measured. Titers were calculated as the reciprocal serum dilution (ID_50_) causing 50% reduction of relative light units. Monoclonal antibodies 084-7D, CB6 and CA1 were used as controls.

*Antibody-dependent cellular cytotoxicity (ADCC) assay*

The ability of the serum antibodies to cross-link between CD16 and spike expressed on cells was used as a proxy for antibody-dependent cellular cytotoxicity (ADCC). To express cell surface spike, HEK 293T cells were transfected with SARS-CoV-2 D614G WT-expressing plasmids and incubated at 37°C for 2 days. Spike-expressing cells were then incubated with serum at a 1:100 final dilution in RPMI medium, 10% fetal bovine serum (FBS), and 1% penicillin-streptomycin for 1 hour at 37°C. Jurkat-Lucia NFAT-CD16 cells (Invitrogen) were added to the reaction and incubated for a further 24 hours at 37°C with 10% CO_2_. Signal was read on a luminometer by adding 20 μl of supernatant and 50 μl of QUANTI-Luc secreted luciferase to white 96-well plates. CR3022, P2B-2F6 and Palivizumab served as controls.

*SARS-CoV-2 spike enzyme linked immunosorbent assay (ELISA)*

2 μg/ml of the D614G spike protein was used to coat 96-well, high-binding plates and incubated overnight at 4°C. The plates were incubated in a blocking buffer consisting of 5% skimmed milk powder, 0.05% Tween 20, 1x PBS. Serum samples were diluted to a 1:100 starting dilution followed by a series of 3-fold serial dilutions. Secondary antibody was diluted to 1:3000 in blocking buffer and added to the plates followed by TMB substrate (Thermofisher Scientific). Upon stopping the reaction with 1 M H_2_SO_4_, absorbance was measured at a 450nm wavelength. Monoclonal antibodies CR3022 and palivizumab were used as controls.

*SARS-CoV-2 nucleocapsid enzyme linked immunosorbent assay (ELISA)*

2 ug/ml of nucleocapsid protein (BioTech Africa; Catalogue number: BA25-P) was used to coat 96-well, high-binding plates and incubated overnight at 4 °C. The plates were incubated in a blocking buffer made up of 1x PBS, 5% skimmed milk powder, 0.05% Tween 20. Serum samples were diluted to a 1:100 dilution in blocking buffer and added to the plates as a single dilution. Secondary antibody was diluted to 1:3000 in blocking buffer and added to the plates followed by TMB substrate (Thermofisher Scientific). Upon stopping the reaction with 1 M H_2_SO_4_, absorbance was measured at a 450nm wavelength. Monoclonal antibodies 1A6 and palivizumab were used as controls.

*Measurement of antigen-specific T cells using flow cytometry*

T cell responses to SARS-CoV2 spike were measured as previously described (PMID: 35102311). Briefly, cryopreserved PBMC were thawed, washed and rested in RPMI 1640 (Sigma-Aldrich, St Louis, MO, USA) containing 10% heat-inactivated foetal calf serum for 4 hours prior to stimulation. PBMC were seeded in a 96-well V-bottom plate at ~2 x 10^6^ PBMC per well and stimulated with a commercial ancestral SARS-CoV-2 spike (S) pool (1 µg/mL, Miltenyi Biotec, Surrey, UK) or variant spike mega pools (15 mers with 10-aa overlap) spanning the entire S of the ancestral, Omicron BA.1 and XBB.1 variants (1 µg/mL). All stimulations were performed in the presence of Brefeldin A (10 µg/mL, Sigma-Aldrich) and co-stimulatory antibodies against CD28 (clone 28.2) and CD49d (clone L25) (1 µg/mL each; BD Biosciences, San Jose, CA, USA). As a negative control, PBMC were incubated with co-stimulatory antibodies, Brefeldin A and an equimolar amount of DMSO. After 16 hours of stimulation, cells were washed, stained with LIVE/DEAD™ Fixable Near-IR Stain (Invitrogen, Carlsbad, CA, USA) and subsequently surface stained with the following antibodies: CD14 APC-Cy7 (HCD14, Biolegend, San Diego, CA, USA), CD19 APC-Cy7 (HIB19, Biolegend), CD4 BV785 (OKT4, Biolegend), CD8 FITC, CD45RA BV570 (HI100, Biolegend), CD27 PE-Cy5 (1A4, Beckman Coulter, Brea, CA, USA). Cells were then fixed and permeabilized using a Cytofix/Cyto perm buffer (BD Biosciences) and stained with CD3 BV650 (OKT3), IFN-γ BV711 (4S.B3), TNF-α PE-Cy7 (Mab11) and IL-2 PE/Dazzle^™^ 594 (MQ1-17H12) from Biolegend. Finally, cells were washed and fixed in CellFIX (BD Biosciences). Samples were acquired on a BD Fortessa flow cytometer and analyzed using FlowJo (v10.8.1 FlowJo LLC, Ashland, OR, USA). Results are expressed as the frequency of total memory CD4 or CD8 T cells expressing IFN-γ, TNF-α or IL-2. Due to high TNF-α backgrounds, cells producing TNF-α alone were excluded from the analysis. All data are presented after background subtraction.
