# Supplementary material for: Safety, effectiveness and immunogenicity of heterologous mRNA-1273 boost after prime with Ad26.COV2.S among healthcare workers in South Africa: The single-arm, open-label, phase 3 SHERPA study
Source: PLOS Glob Public Health. 2024 Dec 5;4(12):e0003260. doi: 10.1371/journal.pgph.0003260 (PMC11620404; doi:10.1371/journal.pgph.0003260)
Supplement: Attachment — Submitted filename: Nyariki_comments_2024.docx [file pgph.0003260.s019.docx]

Garrett et al. present extensive observational data assessing the Safety, Effectiveness, and Immunogenicity of heterologous mRNA-1273 Boost following Ad26.COV2.S Prime among Healthcare Workers in South Africa: the single-arm, open-label, Phase 3 SHERPA Study. While the manuscript reveals significant results which is very important for health. Significant revisions and some issues needs to be addressed as highlighted below for the manuscript to meet publishable standards.

**Title:**

The title effectively communicates the scope, context, and key outcomes of the study however, the title is quite lengthy, which may make it harder for readers to quickly grasp the main focus of the study

Authors: Why too many authors (35)?

**Abstract:**

The abstract is well written, though some issues should be addressed

In line 71 SHERPA should be written full in its first appearance

In line 97 what are the reactogenicity events the authors are referring to? This should be stated

In line 100 the authors should be specific by mentioning the specific antibodies and immune responses that was observed, generalization cannot do justice here.

**Introduction:**

In line 111 instead of stating 2 years let the authors state down specific years (e.g 2020-2021) to bring more clarity

**Materials and Methods**

In line 183 the immediate reactogenicity events should be mentioned

In line 190-201 the authors should describe briefly the procedures for the assays mentioned.

The ethical approval number should be stated by authors

Was sample size determination considered in this study considering the fact that the study involved 501230 Sisonke participants? The authors should shade light on this.

**Results:**

In line 292-293. The author states that “The majority (79.3%) of SHERPA participants were female and median age was 41 years (IQR 35–48)”. However, the 20.7% is not mentioned including the median age. These results for men should be equally described.

In line 408 “ADCC had smaller but significant increases post-boost”. This statement is confusing it is either there is statistical significance or not…but having smaller and significant in the same sentence is misleading. The authors should revise this statement

In Line 453-457 the sentence is too long and redundant, thus the authors should revise it to bring clarity

The use of the Ad26.COV2.S vaccine, is linked with thrombosis and thrombocytopenia syndrome, the authors should at least provide some results that touches in any of these two

**Discussion:**

The discussion section effectively synthesizes the study's results, placing them within the context of current knowledge. However, the authors should address the implications for practice, policy, and future research.

Whereas it provides a balanced interpretation of the findings the authors have failed to acknowledge the limitations and thus this study has not clearly suggested directions for further investigation.

Nevertheless, the overall, the discussion contributes valuable insights into the evolving landscape of COVID-19 vaccination strategies, particularly in diverse and high-risk populations.
