# Supplementary material for: Safety, effectiveness and immunogenicity of heterologous mRNA-1273 boost after prime with Ad26.COV2.S among healthcare workers in South Africa: The single-arm, open-label, phase 3 SHERPA study
Source: PLOS Glob Public Health. 2024 Dec 5;4(12):e0003260. doi: 10.1371/journal.pgph.0003260 (PMC11620404; doi:10.1371/journal.pgph.0003260)
Supplement: Attachment — Submitted filename: Response to Reviewers.docx [file pgph.0003260.s020.docx]

20 August 2024

Abram L. Wagner, PhD, MPH
Academic Editor
PLOS Global Public Health

Dear Dr Wagner,

**Re: Response to Reviewers' Comments for manuscript: PGPH-D-24-00923**

**Manuscript Title:** Safety, Effectiveness and Immunogenicity of heterologous mRNA-1273 Boost after Prime with Ad26.COV2.S among Healthcare Workers in South Africa: the single-arm, open-label, Phase 3 SHERPA Study

We are grateful to you and the reviewers for the comments on our manuscript and for giving us the opportunity to revise and re-submit an improved version of the manuscript for consideration. We have considered all the comments and have updated the manuscript accordingly.

Please find below a point-by-point response to each comment by the Editor and Reviewers with the changes to the manuscript highlighted in **bold** and deleted text in ~~strikethrough~~ format. Pages and line numbers correspond to the tracked changed version of the revised manuscript.

Yours sincerely,

Professor Nigel Garrett

Centre for the AIDS Programme of Research in South Africa (CAPRISA)

Doris Duke Medical Research Institute, University of KwaZulu-Natal

Congella, Durban 4013, South Africa.

Telephone: +27 31 655 0518

E-mail: nigel.garrett@caprisa.org

**Editor’s comments:**

**Comment 1:** Please review your reference list to ensure that it is complete and correct. If you have cited papers that have been retracted, please include the rationale for doing so in the manuscript text, or remove these references and replace them with relevant current references. Any changes to the reference list should be mentioned in the rebuttal letter that accompanies your revised manuscript. If you need to cite a retracted article, indicate the article’s retracted status in the References list and also include a citation and full reference for the retraction notice.

**Response:** Thank you! We have carefully reviewed the reference list and have provided additional information for 2 references.

**Comment 2:** We have noticed that you have uploaded Supporting Information files, but you have not included a list of legends. Please add a full list of legends for your Supporting Information files after the references list.

**Response:** Thank you. The full list of legends for the Supporting Information files have been added after the references list.

**Comment 3:** In the online submission form, you indicated that "Considering this is a clinical trial, universal access to data may not be possible to protect clinical trial participants. However, SHERPA data will be made available by the authors upon reasonable request".

All PLOS journals now require all data underlying the findings described in their manuscript to be freely available to other researchers, either 
1. In a public repository, 
2. Within the manuscript itself, or 
3. Uploaded as supplementary information.

This policy applies to all data except where public deposition would breach compliance with the protocol approved by your research ethics board. If your data cannot be made publicly available for ethical or legal reasons (e.g., public availability would compromise patient privacy), please explain your reasons by return email and your exemption request will be escalated to the editor for approval. Your exemption request will be handled independently and will not hold up the peer review process, but will need to be resolved should your manuscript be accepted for publication. One of the Editorial team will then be in touch if there are any issues.

**Response:** The Sherpa trial manuscript includes different datasets for safety, immunogenicity and effectiveness data. We are happy to work with the Editorial team to provide public access to this data.

**Reviewers' comments:**

**Reviewer's Responses to Questions**

1. Does this manuscript meet PLOS Global Public Health’s [publication criteria](https://url.za.m.mimecastprotect.com/s/b9aTCJZKVAIQzpBkfGEtcE)? Is the manuscript technically sound, and do the data support the conclusions? The manuscript must describe methodologically and ethically rigorous research with conclusions that are appropriately drawn based on the data presented.

Reviewer #1: Yes

Reviewer #2: Yes

Reviewer #3: Yes

Reviewer #4: Yes

2. Has the statistical analysis been performed appropriately and rigorously?

Reviewer #1: Yes

Reviewer #2: Yes

Reviewer #3: Yes

Reviewer #4: Yes

3. Have the authors made all data underlying the findings in their manuscript fully available (please refer to the Data Availability Statement at the start of the manuscript PDF file)?
The [PLOS Data policy](https://url.za.m.mimecastprotect.com/s/0n0ACKO7WBFrw8DQs3ELeC) requires authors to make all data underlying the findings described in their manuscript fully available without restriction, with rare exception. The data should be provided as part of the manuscript or its supporting information, or deposited to a public repository. For example, in addition to summary statistics, the data points behind means, medians and variance measures should be available. If there are restrictions on publicly sharing data—e.g. participant privacy or use of data from a third party—those must be specified.

Reviewer #1: Yes

Reviewer #2: Yes

Reviewer #3: Yes

Reviewer #4: Yes

4. Is the manuscript presented in an intelligible fashion and written in standard English?
PLOS Global Public Health does not copyedit accepted manuscripts, so the language in submitted articles must be clear, correct, and unambiguous. Any typographical or grammatical errors should be corrected at revision, so please note any specific errors here.

Reviewer #1: Yes

Reviewer #2: Yes

Reviewer #3: Yes

Reviewer #4: Yes

**Response:** We thank the Reviewers for these responses.

**Reviewer 1:**

**Comment 1:** The participants of the study have been selected based on few criteria. What are those criteria?

**Response:** Thank you for this comment. All inclusion and exclusion criteria are presented in Supplementary Table 2 entitled: ‘Eligibility criteria for the SHERPA trial’. This is referred to on page 7, lines 169-177 of the track changed manuscript.

**Comment 2:** The participants immune status was not assessed. Please Explain why?

**Response:** All participants had a plasma sample taken to test for SARS-CoV-2 serology before vaccination to assess SARS-CoV-2 immune status. This is explained on p.7, l. 183. HIV immune status, e.g. CD4+ T-cell counts were not specifically collected in this Covid-19 vaccine trial.

**Comment 3:** The ELISA was applied to detect Ig status. Does the ELISA kit validated?

**Response:** Thank you for this comment. We confirm that the ELISA kit to test for SARS-CoV-2 serology at baseline we the validated assay run on the Architect platform by Abbott, Chicago, IL, USA. We have added this information to the methods section, p. 7, l. 184. The word ‘validated’ was also added to the immunogenicity sub-study section, p. 8, l. 200.

**Comment 4:** The process of antigen preparation is not mentioned here. Please write in details.

**Response:** This study did not prepare any antigens for the routine testing. As mentioned above, a validated assay was used to do the SARS-CoV-2 testing. More details about the immunogenicity sub-study methods are provided in the supplementary materials, now referenced at the end of the manuscript.

**Comment 5:** The exposure history of exposure is not mentioned here. Please describe this.

**Response:** Thank you for this comment. We assume that the reviewer is referring to a history of prior SARS-CoV-2 exposure. This was measured through antibody testing at enrolment.

**Comment 6:** How did the authors measure the T-Cell count? This is missing in the methodology section.

**Response:** Please note that we did not conduct routine CD4+ T-cell count testing in the SHERPA trial. However, specific T-cell assays were conducted in the immunogenicity sub-study. The Methodology for T-cell measurements is included in the Supplementary Material. It is pasted below for ease of reference and has been cross referenced on p.8, l. 209 of the manuscript:

*‘Measurement of antigen-specific T cells using flow cytometry*

*T cell responses to SARS-CoV2 spike were measured as previously described (PMID: 35102311). Briefly, cryopreserved PBMC were thawed, washed and rested in RPMI 1640 (Sigma-Aldrich, St Louis, MO, USA) containing 10% heat-inactivated foetal calf serum for 4 hours prior to stimulation. PBMC were seeded in a 96-well V-bottom plate at ~2 x 10^6^ PBMC per well and stimulated with a commercial ancestral SARS-CoV-2 spike (S) pool (1 µg/mL, Miltenyi Biotec, Surrey, UK) or variant spike mega pools (15 mers with 10-aa overlap) spanning the entire S of the ancestral, Omicron BA.1 and XBB.1 variants (1 µg/mL). All stimulations were performed in the presence of Brefeldin A (10 µg/mL, Sigma-Aldrich) and co-stimulatory antibodies against CD28 (clone 28.2) and CD49d (clone L25) (1 µg/mL each; BD Biosciences, San Jose, CA, USA). As a negative control, PBMC were incubated with co-stimulatory antibodies, Brefeldin A and an equimolar amount of DMSO. After 16 hours of stimulation, cells were washed, stained with LIVE/DEAD™ Fixable Near-IR Stain (Invitrogen, Carlsbad, CA, USA) and subsequently surface stained with the following antibodies: CD14 APC-Cy7 (HCD14, Biolegend, San Diego, CA, USA), CD19 APC-Cy7 (HIB19, Biolegend), CD4 BV785 (OKT4, Biolegend), CD8 FITC, CD45RA BV570 (HI100, Biolegend), CD27 PE-Cy5 (1A4, Beckman Coulter, Brea, CA, USA). Cells were then fixed and permeabilized using a Cytofix/Cyto perm buffer (BD Biosciences) and stained with CD3 BV650 (OKT3), IFN-γ BV711 (4S.B3), TNF-α PE-Cy7 (Mab11) and IL-2 PE/Dazzle^™^ 594 (MQ1-17H12) from Biolegend. Finally, cells were washed and fixed in CellFIX (BD Biosciences). Samples were acquired on a BD Fortessa flow cytometer and analyzed using FlowJo (v10.8.1 FlowJo LLC, Ashland, OR, USA). Results are expressed as the frequency of total memory CD4 or CD8 T cells expressing IFN-γ, TNF-α or IL-2. Due to high TNF-α backgrounds, cells producing TNF-α alone were excluded from the analysis. All data are presented after background subtraction.’* 

**Comment 7:** The results section is missing the AEs or SAEs. Please mention.

**Response:** The adverse events (AEs) and serious adverse events (SAEs) are mentioned on p.16, l. 383-387 of the manuscript and supplementary table 10.

**Reviewer 2:**

**Comment 1:** The manuscript is generally well written except for a few grammatical errors
1.The abstract and introduction are difficult to follow. The authors need to make it understandable to the reader,

**Response:** Thank you for this comment. We have reviewed the manuscript for grammatical errors and corrected these where found. We have made changes to the abstract and introduction to make it easier for the reader. Please see for example in the Abstract, p.4, l. 79-81.

**Comment 2:** Abbreviations should be defined when first used e.g. mcg

**Response:** We have reviewed all abbreviations and checked that all are spelled out in full the first time they are used.

**Reviewer 3:**

**Comment 1:** Garrett et al. present extensive observational data assessing the Safety, Effectiveness, and Immunogenicity of heterologous mRNA-1273 Boost following Ad26.COV2.S Prime among Healthcare Workers in South Africa: the single-arm, open-label, Phase 3 SHERPA Study. While the manuscript reveals significant results which is very important for health. Significant revisions and some issues needs to be addressed as highlighted below for the manuscript to meet publishable standards.

Title: The title effectively communicates the scope, context, and key outcomes of the study however, the title is quite lengthy, which may make it harder for readers to quickly grasp the main focus of the study

Authors: Why too many authors (35)?

**Response:** Thank you for this comment. The large SHERPA trial was conducted at 30 clinical research sites in South Africa. The principal investigators of each of these sites were included as co-author considering the large number of participants at each site. In addition, the author list includes key protocol team members, statisticians and laboratory scientists who all made significant contributions to the trial conduct, analysis and data interpretation. The author number was also checked with the Editorial team of the journal. We believe that the title is informative, as concise as possible, and includes the study design.

**Comment 2:** Abstract: The abstract is well written, though some issues should be addressed. In line 71 SHERPA should be written full in its first appearance

**Response:** Thank you - this has been written in full the first time it is mentioned in the abstract (p. 4, l. 81).

**Comment 3:** In line 97 what are the reactogenicity events the authors are referring to? This should be stated

**Response:** Thank you for asking for these details. We have added more details to the abstract, p. 4, l. 98-105.

*‘Of 11798 participants in the safety analysis,* ***228 (1.9%) participants reported 575 reactogenicity events within 7 days of the booster (most commonly injection site pain, malaise, myalgia, swelling, induration and fever)~~71 (2.3%) reported a reactogenicity event or unsolicited AE~~,*** *more among those with prior SARS-CoV-2 infections (adjusted odds ratio [aOR] 2.03, 95%CI 1.59-2.59) and less among people living with HIV (PLWH) (aOR 0.49, 95%CI 0.34-0.69).* ***There were 115 unsolicited adverse events (AEs) within 28 days of vaccination.*** *No related serious AEs were reported.’*

More details were already included in the Results section of the manuscript under the safety heading (copied and pasted below).

*‘Of 11798 SHERPA participants included in the safety analysis, 228 (1.9%) participants reported 575 reactogenicity events within 7 days after the mRNA-1273 booster. Of these, 230/575 (40%) were local reactogenicity events including 97/230 (42.2%) injection site pain, 69/230 (30%) swelling, 32/230 (13.9%) induration and 32/230 (13.9%) erythema events. A total of 345 systemic reactogenicity events were reported including 123 (35.7%) malaise and/or myalgia, 120 (34.8%) headache, 66 (19.1%) fever, 17 (4.9%) chills, 10 (2.9%) nausea, 5 (1.4%) vomiting and 4 (1.2%) arthralgia. All reported local and systemic reactogenicity were mild or moderate (grade 1 or 2) and all resolved without sequalae.*

*Unsolicited AEs collected for 28 days after vaccination and those meeting SAE/AESI criteria collected until the end of study participation were reported by 98 (0.8%) participants, reporting a total of 115 events. Of these events, 17 events met SAE criteria. Three of the 17 SAEs also met AESI criteria, the other 4 AESIs were grade 1 and 2 anosmia and ageusia, which resolved without sequalae. No related SAEs were reported during the study.*

*More reactogenicity events were reported by participants with prior SARS-CoV-2 infection (3.6% vs 1.8%), HIV-negative status (2.7% vs 1.2%) and those who received two compared to one prior Ad26.COV2.S dose (2.6% vs 2.0%). In a logistic regression model adjusting for age and sex, a prior SARS-CoV-2 infection (adjusted odds ratio [aOR] =2.03, 95%CI 1.59-2.59) and an HIV positive status (aOR=0.49, 95%CI 0.34-0.69) were associated with local/systemic reactions, but not having received two versus one Ad26.COV2.S dose before the mRNA-1273 booster (aOR=1.26, 95%CI 0.99-1.62), Supplementary Table 9.’*

**Comment 4:** In line 100 the authors should be specific by mentioning the specific antibodies and immune responses that was observed, generalization cannot do justice here.

**Response:** Considering the immunology analysis was not the primary objective of the study, we decided to only summarize these results in the abstract. However, we have specified the text to make it clearer and the results section covers this comprehensively: p. 5, l. 106: *‘In an immunogenicity sub-study, mRNA-1273 increased* ***binding and*** ***neutralizing*** *antibody ~~functions~~* ***titres*** *and* ***spike-specific*** *T-cell responses 4 weeks after boosting regardless of the number of prior Ad26.COV2.S doses, or HIV status, and generated Omicron spike-specific cross-reactive responses.’*

**Comment 5:** Introduction: In line 111 instead of stating 2 years let the authors state down specific years (e.g 2020-2021) to bring more clarity

**Response:** Thank you – the years ***‘2020-2022’*** have now been added.

**Comment 6:** Materials and Methods: In line 183 the immediate reactogenicity events should be mentioned

**Response:** The reviewer is referring to line 183, which is in the methods section. The immediate reactogenicity events are properly described in the results section of the manuscript. Please see the safety section (p. 14, l. 373-381).

**Comment 7:** In line 190-201 the authors should describe briefly the procedures for the assays mentioned.

**Response:** Considering that the methods section is already long, and the immunogenicity sub-study is not the primary objective of the study, we decided to summarize the immunology assays on p. 8, l. 198–209 for the general reader. However, if a reader is more interested in this topic, we have provided more details in the supplementary material section. We have therefore added a note to make the reader aware of this option in l. 209. Furthermore, we have now added the supplementary text on immunology methods as a heading to the end of this manuscript.

**Comment 8:** The ethical approval number should be stated by authors.

**Response:** We have included the regulatory and ethical approval numbers for the SHERPA trial of the regulator, SAHPRA, and the 7 ethics committees in South Africa (see p.10-11, l. 271-279).

**Comment 9:** Was sample size determination considered in this study considering the fact that the study involved 501230 Sisonke participants? The authors should shade light on this.

**Response:** Although this was a pragmatic implementation trial, sample size and power calculations were considered. Given the limited vaccine doses available, the target sample size in the SHERPA study was 15000 mRNA vaccine recipients. Assuming an attack rate of 0.3% (i.e. COVID-19 hospitalizations or deaths) observed among vaccinated individuals in the larger Sisonke cohort. Enrolling a total of 10332 mRNA vaccine recipients and 41328 non-mRNA recipients in a ratio of 1:4 provided us with 80% power to detect a relative vaccine effectiveness (rVE) of 45% or greater. The power calculations were based on a log-rank test. Furthermore, with an attack rate of 5%, a sample size of 7652 mRNA boost recipients and 30608 non-mRNA boost recipients confers more than 80% power to detect a rVE of 15% or greater against any COVID-19 infection.

To ensure that the highest number of endpoints were included in the analyses, a decision was made to conduct an analysis that includes all Sisonke participants (N=477 000), 15000 of whom were mRNA vaccine boosted. Under this approach (referred to as the adjusted cohort analyses), with the aforementioned group sizes, an attack rate of 0.3%, a rVE of 40% or greater against severe disease (associated with observing 878 events) could be detected with 92% power.

Results from both approaches are presented in the manuscript, with the latter serving as the primary analyses. Further details on this are provided in the study protocol and the statistical analysis plan, which were submitted together with the manuscript to the journal.

**Comment 10:** Results: In line 292-293. The author states that “The majority (79.3%) of SHERPA participants were female and median age was 41 years (IQR 35–48)”. However, the 20.7% is not mentioned including the median age. These results for men should be equally described.

**Response:** Thank you. We have now made this clearer by splitting the sentence. Please note that the median age applied to the overall population.

p.12, l. 302: ***‘****The majority (79.3%) of SHERPA participants were female* ***and 20.7% were male****. The median age was 41 years (IQR 35–48).’*

**Comment 11:** In line 408 “ADCC had smaller but significant increases post-boost”. This statement is confusing it is either there is statistical significance or not…but having smaller and significant in the same sentence is misleading. The authors should revise this statement

**Response:** We have clarified this section as suggested.

p.18, l. 418-419: *‘Binding titers significantly increased up to 6-fold 4 weeks post-boost (Fig. 2D,E), ~~while~~* ***and*** *ADCC ~~had smaller but~~* ***also increased significantly*** *post-boost (Fig. 2F,G).’*

**Comment 12:** In Line 453-457 the sentence is too long and redundant, thus the authors should revise it to bring clarity

**Response:** Please note that this section is not manuscript text, but the Figure 3 legend. We believe this is an appropriate legend. Apologies if this was not clear to the Reviewer, but it was a journal submission requirement to insert the Figure legends into the manuscript text.

**Comment 13:** The use of the Ad26.COV2.S vaccine, is linked with thrombosis and thrombocytopenia syndrome, the authors should at least provide some results that touches in any of these two

**Response:** In the SHERPA study, where mRNA-1273 not Ad26.COV.S vaccines was administered, no participants experienced thrombosis and thrombocytopaenia syndrome. However, we previously reported on this adverse event as part of the Sisonke trial (please see citation 3 and other manuscripts by this research team).

**Comment 14:** Discussion: The discussion section effectively synthesizes the study's results, placing them within the context of current knowledge. However, the authors should address the implications for practice, policy, and future research.

**Response:** Thank you – we have revised the discussion section to further address the implications for practice, policy, and future research.

p.23, l. 571-582: ‘*This* ***has policy and programming complications*** *~~is important~~* ***as homologous boosting and*** *variant vaccines may not be accessible in these settings. In conclusion, the SHERPA trial showed a high rVE of the mRNA-1273 booster during the Omicron wave among healthcare workers who had received one or two previous doses of Ad26.COV2.S. The heterologous booster was safe and well tolerated, immunogenic and effective in a LMIC with high HIV prevalence.* ***This study provides in principle support for transitioning from homologous to heterologous boosters with Ad26 and mRNA platforms in the event of future pandemics where vaccine availability may be limited. Additionally, it provides evidence that variant viruses do not always need variant vaccines. While this is especially pertinent to LMIC settings, it does not preclude the need to monitor variants and the neutralisation capacity of parent vaccines before pandemic vaccination policies are finalised and implemented.’***

**Comment 15:** Whereas it provides a balanced interpretation of the findings the authors have failed to acknowledge the limitations and thus this study has not clearly suggested directions for further investigation. Nevertheless, the overall, the discussion contributes valuable insights into the evolving landscape of COVID-19 vaccination strategies, particularly in diverse and high-risk populations.

**Response:** We thank the Reviewer on the positive feedback on our study relevance. We have expanded the limitation section in the discussion section of the manuscript, p.22/23, l. 552-565:

*‘SHERPA had some limitations. First, it was a single arm trial nested in the larger Sisonke trial. As in Sisonke, we relied on routinely collected data to ascertain COVID-19 endpoints. However, this applied to both mRNA-1273 boosted and unboosted study groups, therefore reducing the potential for reporting bias. Second, we observed too few severe COVID-19 endpoints to allow for a well-powered assessment of rVE against severe disease. Contributing factors could have been that SHERPA was conducted during the Omicron wave, when generally less severe disease was reported^28^, South Africa has a high SARS-CoV-2 seroprevalence of up to 80%^29^, and all participants had at least received one dose of Ad26.COV2.S previously.* ***Third, we presented short to medium term rVE results, and were not able to assess the longer term protection of the mRNA-1273 booster due to national changes in case reporting.*** *Finally, we were unable to compare pregnancy outcomes in the SHERPA study with the larger Sisonke trial population, because pregnant women were initially not eligible for Sisonke, and pregnancy outcomes were not systematically collected.’*

**Reviewer 4:**

**Comment 1:** The study design leveraging data from the SHERPA trial nested within the Sisonke study provides robust evidence on the effectiveness and safety of the mRNA-1273 booster in individuals previously vaccinated with Ad26.COV2.S.
The large sample size and comprehensive data collection allow for detailed subgroup analyses, particularly regarding different doses of Ad26.COV2.S and participant demographics (e.g., HIV status).
The inclusion of both unadjusted and adjusted analyses for vaccine effectiveness against SARS-CoV-2 infections adds depth to the findings, enhancing the reliability of the reported results.

**Response:** Many thanks for highlighting the strengths of our study.

**Comment 2:** Limitations
While the study provides valuable insights into the effectiveness of the mRNA-1273 booster, there are limitations in the follow-up period, especially regarding the duration of immune response post-booster. Longer-term data would strengthen the assessment of sustained protection.
The exclusion criteria, such as participants receiving a third Ad26.COV2.S or BNT162b2 booster, could potentially influence the generalizability of the findings, particularly in settings where multiple boosters are administered.
The interpretation of vaccine effectiveness against severe COVID-19 outcomes is limited by the small number of events, particularly in the SHERPA cohort, which affects the precision of these estimates.

**Response:** Thank you for these comments. We agree with your assessment and have expanded the limitation section in the Discussion, see p.23, l. 560-562 and our response to Comment 15 by Reviewer 3 above.

**Comment 3:** Recommendations
Provide additional discussion on the implications of the findings in the context of emerging variants, especially Omicron, given the breakthrough infections observed during this period.

Consider discussing the potential implications of these findings on vaccine policy and public health strategies, particularly in populations with varying levels of prior exposure to COVID-19 and different vaccine schedules.

**Response:** Many thanks you for these excellent suggestions. More information has now been added at the end of the discussion section (see p.23, l. 576-582). Please see response to Comment 14 by Reviewer 3 above, for the added text.

**Comment 5:** Strengthen the conclusions by addressing the limitations related to data completeness, particularly in subgroup analyses, and provide insights into future research directions, such as the need for continued monitoring of vaccine durability and effectiveness against evolving variants.

**Response:** Thank you for this comment. The following sentence has been added at the end of the discussion, p 24, l.582-583:

***‘There is a need for future research to ensure continued monitoring of vaccine durability and effectiveness against evolving variants.’***

**Comment 6:** Overall Impact
This study significantly contributes to the evolving literature on COVID-19 vaccine effectiveness and safety, particularly in the context of heterologous booster strategies.
The findings are timely and relevant for informing vaccination strategies globally, especially in populations with initial Ad26.COV2.S vaccinations, including considerations for special populations such as individuals living with HIV.

**Response:** We appreciate your review and the positive feedback.
