# Supplementary material for: Safety, effectiveness and immunogenicity of heterologous mRNA-1273 boost after prime with Ad26.COV2.S among healthcare workers in South Africa: The single-arm, open-label, phase 3 SHERPA study
Source: PLOS Glob Public Health. 2024 Dec 5;4(12):e0003260. doi: 10.1371/journal.pgph.0003260 (PMC11620404; doi:10.1371/journal.pgph.0003260)
Supplement: Attachment — Submitted filename: Response to Statistical Reviewer.docx [file pgph.0003260.s021.docx]

12 October 2024

**Re: Response to Statistical Reviewer's Comments for manuscript: PGPH-D-24-00923**

**Manuscript Title:** Safety, Effectiveness and Immunogenicity of heterologous mRNA-1273 Boost after Prime with Ad26.COV2.S among Healthcare Workers in South Africa: the single-arm, open-label, Phase 3 SHERPA Study

We are grateful for the statistical review of our manuscript, and fully acknowledge some of the study design limitations of the single arm trial that are highlighted by Reviewer 5. We have expanded the limitation section in the discussion section to cover these points in more detail.

Please find below our responses to each comment of Reviewer 5. Changes to the manuscript are highlighted in **bold**. Pages and line numbers correspond to the tracked changed version of the second revision of the manuscript.

**Statistical Reviewer's comments:**

The study investigates the safety and effectiveness of a heterologous mRNA-1273 booster in healthcare workers primed with Ad26.COV2.S, making it a clear piece of primary research. The methodology is well-defined with the use of a single-arm, open-label, phase 3 design nested within a larger trial (Sisonke).

The are some comments as listed below:

**Comment 1:** The lack of randomization is the most significant limitation when comparing the SHERPA study to the unboosted Sisonke cohort. Randomization is critical for ensuring that treatment groups are comparable in terms of both observed and unobserved factors, thereby minimizing confounding and selection bias. Since SHERPA lacks randomization, the comparison with the unboosted Sisonke participants comes with several inherent risks.

- Selection Bias: Without randomization, the SHERPA participants (those who received the mRNA-1273 booster) may differ systematically from the unboosted Sisonke participants in ways that are not controlled for. For instance, participants who opted to receive the booster might be more health-conscious or have different risk factors, comorbidities, or exposure risks than those who did not get boosted.

- Even though statistical adjustments (e.g., controlling for age, sex, prior infection) are used in the Cox model, unmeasured confounders (e.g., occupation type, risk behaviors, healthcare access) could bias the results. These factors would likely have been balanced if randomization were used.

- Confounding Variables: In non-randomized studies, confounding variables—both measured and unmeasured—can skew the results. The SHERPA study adjusts for several confounders like prior SARS-CoV-2 infection, comorbidities, and geographic location, but the absence of randomization leaves room for residual confounding.

For example, SHERPA participants could have a different exposure to the virus compared to unboosted Sisonke participants, especially if the timing of vaccination and circulating variants differed. Such confounding variables may not be adequately captured by the existing adjustments.

- Immortal Time Bias: Immortal time bias can arise when participants in one group (e.g., SHERPA) are considered immune to the outcome (infection, severe disease) for a certain period. In this case, the time-varying nature of the mRNA-1273 booster is accounted for in the model, but without randomization, there’s still a risk that some bias persists, particularly in how the time frames for receiving the vaccine differ between groups.

- Baseline Differences: Randomization ensures that groups start with comparable baseline characteristics. In SHERPA’s comparison with Sisonke, the lack of randomization means there could be significant differences at baseline, such as the time since initial vaccination or underlying health conditions, which could affect the vaccine effectiveness estimates.

**Response:** We thank the reviewer for this comment and acknowledge the many statistical challenges and potential biases in design and analysis of real-world effectiveness studies. A challenge in effectiveness designs compared to efficacy trials is that randomization is not feasible. As pointed out by the reviewer, and as recommended by the WHO (https://www.who.int/publications/i/item/WHO-2019-nCoV-vaccine_effectiveness-measurement-2021.1), we have applied various approaches in an effort to reduce potential biases including the adjustment of measured confounders, as well as an additional approach of the matched cohort design. We acknowledge that randomization did not occur and that residual biases may still be present in our analysis. We have now highlighted this further in the study limitation section of the discussion.

Page 22, line 545: *‘First, it was a single arm trial nested in the larger Sisonke trial,* ***and participants were not randomized.’***

Page 22, line 554 – 557: ***‘Fourth, while the adjusted cohort and matched cohort approaches seek to emulate a clinical trial from observational data, residual bias emanating from the impact of unmeasured confounders or selection bias may still remain and therefore results should be carefully interpreted.’***

**Comment 2:** Statistical Techniques Attempt to Mitigate But Not Replace Randomization:

- Cox Proportional Hazards with Time-Varying Covariates and Matched Cohort Analyses are used in the study to attempt to balance the groups and reduce bias. While these methods can help control for measured confounders, they cannot account for unmeasured variables, leaving the comparison vulnerable to bias.

- Techniques like propensity score matching (PSM), inverse probability of treatment weighting (IPTW), or stratification based on propensity scores can improve the balance of measured confounders but still fall short of the robustness that randomization would provide. These methods adjust for known covariates but cannot guarantee that all relevant confounders are accounted for.

The above points in 1) and 2) need to be addressed. The lack of randomization is a critical limitation that cannot be fully addressed through statistical adjustments alone. While techniques like Cox models and propensity score matching can help to balance observed covariates, they cannot compensate for unmeasured confounders and selection bias. As a result, the comparison between SHERPA and the Sisonke cohort must be interpreted with caution. The absence of randomization reduces the strength of the causal claims and introduces uncertainty about whether the observed differences in outcomes are truly due to the mRNA-1273 booster.

For a more robust and reliable comparison, a randomized controlled trial (RCT) or at least a well-designed randomized arm within the SHERPA study would have been ideal.

**Response 2:** The reviewer is correct in noting that the two statistical approaches outlined (covariate adjustment and matching) which have been applied cannot accommodate or account for unmeasured confounders. We have further emphasized this point in the discussion by adding a following text:

Page 22, line 554 – 557: ‘***Fourth, while the adjusted cohort and matched cohort approaches seek to emulate a clinical trial from observational data, residual bias emanating from the impact of unmeasured confounders or selection bias may still remain and therefore results should be carefully interpreted.’***
